# The SINA1‐BSD1 Module Regulates Vegetative Growth Involving Gibberellin Biosynthesis in Tomato

**DOI:** 10.1002/advs.202400995

**Published:** 2024-08-27

**Authors:** Yulin Yuan, Youhong Fan, Li Huang, Han Lu, Bowen Tan, Chloe Ramirez, Chao Xia, Xiangli Niu, Sixue Chen, Mingjun Gao, Cankui Zhang, Yongsheng Liu, Fangming Xiao

**Affiliations:** ^1^ Department of Plant Sciences University of Idaho Moscow ID 83844 USA; ^2^ School of Food and Biological Engineering Hefei University of Technology Hefei Anhui 230009 China; ^3^ Ministry of Education Key Laboratory for Biodiversity Science and Ecological Engineering National Observations and Research Station for Wetland Ecosystems of the Yangtze Estuary Institute of Biodiversity Science and Institute of Eco‑Chongming School of Life Sciences Fudan University Shanghai 200433 China; ^4^ Department of Biology University of Mississippi Oxford MS 38677 USA; ^5^ Maize Research Institute Sichuan Agricultural University Chengdu 611130 China; ^6^ Department of Agronomy Purdue Center for Plant Biology Purdue University 915 Mitch Daniels Blvd West Lafayette IN 47907 USA; ^7^ School of Horticulture Anhui Agricultural University Hefei Anhui 230036 China; ^8^ Ministry of Education Key Laboratory for Bio‐resource and Eco‐environment College of Life Science State Key Laboratory of Hydraulics and Mountain River Engineering Sichuan University Chengdu Sichuan 610064 China

**Keywords:** BRG1, BSD1, gibberellin, SINA1, tomato, transcription factor, ubiquitin ligase

## Abstract

In plants, vegetative growth is controlled by synergistic and/or antagonistic effects of many regulatory factors. Here, the authors demonstrate that the ubiquitin ligase seven in absentia1 (SINA1) mammalian BTF2‐like transcription factors, Drosophila synapse‐associated proteins, and yeast DOS2‐like proteins (BSD1) function as a regulatory module to control vegetative growth in tomato via regulation of the production of plant growth hormone gibberellin (GA). SINA1 negatively regulates the protein level of BSD1 through ubiquitin‐proteasome‐mediated degradation, and the transgenic tomato over‐expressing *SINA1* (*SINA1‐OX*) resembles the dwarfism phenotype of the *BSD1*‐knockout (*BSD1‐KO*) tomato plant. BSD1 directly activates expression of the *BSD1‐regulated gene 1* (*BRG1*) via binding to a novel core BBS (standing for BSD1 binding site) binding motif in the *BRG1* promoter. Knockout of *BRG1* (*BRG1‐KO*) in tomato also results in a dwarfism phenotype, suggesting BRG1 plays a positive role in vegetative growth as BSD1 does. Significantly, GA contents are attenuated in transgenic *SINA1‐OX*, *BSD1‐KO*, and *BRG1‐KO* plants exhibiting dwarfism phenotype and exogenous application of bioactive GA_3_ restores their vegetative growth. Moreover, BRG1 is required for the expression of multiple GA biosynthesis genes and BSD1 activates three GA biosynthesis genes promoting GA production. Thus, this study suggests that the SINA1‐BSD1 module controls vegetative growth via direct and indirect regulation of GA biosynthesis in tomato.

## Graphical abstract

This study reveals how plant growth is controlled by a protein regulatory module SINA1‐BSD1. SINA1 is an enzyme that regulates the growth activator BSD1 through protein degradation. This regulation ultimately affects the production of gibberellin, a crucial plant growth hormone. The findings suggest that SINA1 and BSD1 act as a regulatory module, fine‐tuning plant growth by modulating gibberellin biosynthesis.

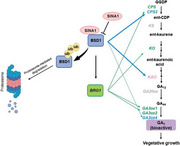

## 1 Introduction

Vegetative growth, including internode elongation that is a main factor determining the overall plant height, is regulated by intricate biochemical and genetic interaction networks in plants.^[^ ^1^ ^]^ Impairment in these regulatory pathways may result in interference with vegetative growth thereby a dwarfism phenotype that is usually associated with abnormal internode elongation caused by alterations in cell number, cell size, or both.^[^ ^1^ ^]^ Up to date, much effort has been made to investigate the molecular basis of the regulation of vegetative growth in plants, particularly focusing on plant hormones that are generally involved in various aspects plant growth and development, among which, gibberellin (GA) is the major plant hormone regulating vegetative growth at an ultra‐low concentration.^[^ ^2^ ^]^ Numerous studies have shown that disruption in the GA biosynthesis or signaling pathway results in a dwarfism phenotype.^[^ ^3^ ^]^ For example, mutations in GA biosynthesis genes such as *KAO* encoding *ent*‐kaurenoic acid oxidase in maize ^[^ ^4^ ^]^, *GA3ox1* encoding the gibberellin 3‐oxidase in *Arabidopsis*,^[^ ^5^ ^]^ and *GA20ox2‐2* encoding the gibberellin 20‐oxidase in rice ^[^ ^6^ ^]^ lead to significant reduction of plant height. Same as the dwarfism phenotype caused by mutations in GA biosynthesis genes, *Arabidopsis* exhibits severe dwarfism when all three homologous genes encoding GA signaling receptor GIBBERELLIN‐INSENSITIVE DWARF1 (GID1) are mutated.^[^ ^7^ ^]^

Transcription factors may act as key regulators of vegetative growth due to their potential activation of or repression on many genes’ expression cooperatively. In rice, several transcription factors have been identified to regulate vegetative growth. Among them, OsMPH1,^[^ ^8^ ^]^ OsDTH8,^[^ ^9^ ^]^ OsMADS57 ^[^ ^10^ ^]^ and OsSGD2 (small grain and dwarf 2) ^[^ ^11^ ^]^ act as positive regulators of vegetative growth, whereas OsAP2/ERF,^[^ ^12^ ^]^ OsWRKY55,^[^ ^13^ ^]^ OsWRKY21,^[^ ^14^ ^]^ OsWRKY36 ^[^ ^15^ ^]^ and OsNAC2 ^[^ ^16^ ^]^ function as negative regulators. In tomato (*Solanum lycopersicum*), the role of a few transcription factors in controlling vegetative growth has been demonstrated. For example, BZR1, a transcription factor involved in the brassinosteroid (BR) signaling pathway, negatively regulates plant height via manipulation of BR‐mediated vegetative growth.^[^ ^17^ ^]^ SlNAP1, a NAC (NAM, ATAF1/2, and CUC2) type transcription factor, negatively affects vegetative growth when constitutively overexpressed in transgenic tomato plants.^[^ ^18^ ^]^ However, genetic manipulation of another NAC transcription factor SlNAC6, either by constitutive overexpression or by RNA interference (RNAi)‐based gene repression, results in significant delay in vegetative growth, likely through affecting abscisic acid (ABA)‐mediated signaling pathway,^[^ ^19^ ^]^ suggesting the complexity of regulation of vegetative growth in tomato.

The ubiquitin‐proteasome system (UPS) is the major intracellular protein degradation system in eukaryotic cells and plays important roles in diverse physiological processes in plants.^[^ ^20^ ^]^ Ubiquitination requires an enzymatic cascade involving the ubiquitin‐activating enzyme (E1), ubiquitin‐conjugating enzyme (E2) and ubiquitin ligase (E3), of which the ubiquitin ligase determines the substrate specificity for UPS. A growing body of evidence has shown that UPS plays important roles in vegetative growth,^[^ ^21^ ^]^ presumably via regulation of the protein levels of the positive or negative regulators (e.g., aforementioned transcription factors involved in vegetative growth), in which regulators are ubiquitinated by ubiquitin ligases for proteasome‐mediated degradation. Seven in absentia (SINA) ubiquitin ligases belong to the RING‐type E3 family and contain an N‐terminal Really Interesting New Gene (RING) domain that binds to E2, followed by two zinc‐fingers and a typical SINA domain devoted to substrate recognition and dimerization.^[^ ^22^ ^]^ Although SINA was originally identified in *Drosophila* with a significant role in photoreceptor differentiation, it has been demonstrated recently that SINA proteins play important roles in many physiological processes in diverse plant species, including development and growth, abiotic stress response, and plant‐microbe interactions.^[^ ^23^, ^24^ ^]^ In tomato, six SINA proteins (termed SINA1‐6) have been identified, and they all possess RING‐dependent E3 ubiquitin ligase activity and exhibit similar preference to E2 ubiquitin‐conjugating enzymes in the ubiquitination reaction.^[^ ^22^ ^]^ The functionality of SINA proteins has been preliminarily investigated. SINA4 plays a positive role in defense signaling, as manifested by elicitation of E3‐dependent hypersensitive response (HR)‐like cell death; the other SINAs are negative regulators of defense signaling and capable to suppress HR cell death. In addition, transgenic tomato plants overexpressing *SINA2* exhibit pale‐green leaf phenotype, suggesting SINA2 regulates chlorophyll level in plant cells, whereas SINA5 plays a role in flower development.^[^ ^22^ ^]^ However, despite it is known that SINA3 plays a role in disease resistance via ubiquitination of the defense‐related transcription factor SlNAC1,^[^ ^25^ ^]^ the mechanistic basis by which SINA2/4/5 regulates the distinct physiological process has not been elucidated.

Recently, we identified a novel BSD1 (mammalian BTF2‐like transcription factors, *Drosophila* synapse‐associated proteins, and yeast DOS2‐like proteins) transcription factor in tomato, which plays an important role in controlling vegetative growth, as manifested by a dwarfism phenotype in the *BSD1 RNA‐interference* (termed *BSD1‐Ri* hereafter) line.^[^ ^26^ ^]^ However, how the BSD1 transcription factor is regulated and what genes are activated by BSD1 in the context of vegetative growth have not been addressed. Significantly, our previous research showed that the *BSD1* gene is ubiquitously expressed in all tested tissues and its expression is not dramatically affected during growth,^[^ ^26^ ^]^ suggesting the BSD1 may be regulated at post‐transcriptional or/and ‐translational level. Here, our further study demonstrates that the BSD1 protein is regulated by the ubiquitin ligase SINA1. BSD1 activates expression of the *BRG1* gene, which encodes a hypothetic protein that is involved in GA‐mediated cell expansion in internodes, by directly binding to a novel binding motif BBS in *BRG1* promoter. In parallel, BSD1 also regulates three BBS‐containing GA biosynthesis genes promoting GA production in internodes.

## 2 Results

### 2.1 BSD1 is Required for Vegetative Growth in Tomato

**Figure 1:**
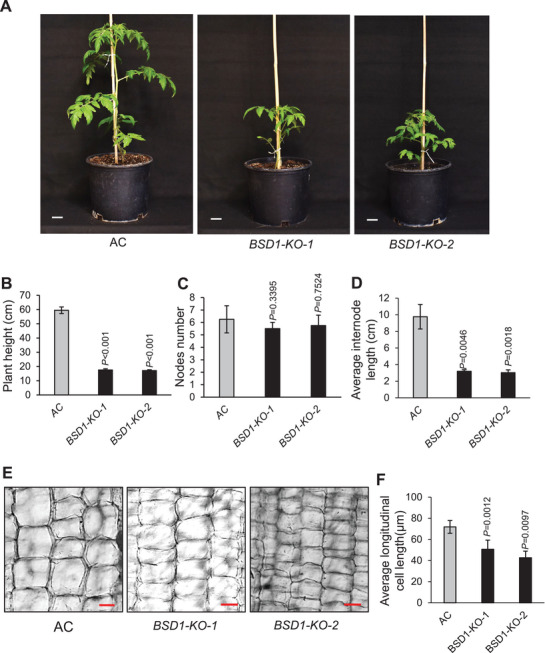
BSD1 positively regulates vegetative growth in tomato. A) The morphologic appearance of representative six‐week‐old transgenic *BSD1‐KO* and non‐transgenic AC plants. Scale bar, 5 cm. B‐D) Statistical analyses of plants’ height B), node number C), and average internode length D) of plants in (A). N = 4. *P* values are indicated in the graph. E) Longitudinal section and staining with toluidine blue for the 3^rd^ internode stems of two‐month‐old AC and *BSD1‐KO* plants. Scale bar, 50 µm. F) Quantification of the longitudinal length of cells in (E). N = 3. *P* values are indicated in the graph. Data are means ± SD; ^*^ *p* < 0.05, two‐tailed *t*‐tests. All experiments have been repeated at least twice with similar results.

Previously we identified a tomato *BSD1* gene encoding a novel transcription factor and knockdown of *BSD1* in transgenic tomato *BSD1* RNAi line *BSD1‐Ri* resulted in significant interference with vegetative growth.^[^ ^26^ ^]^ To determine the role of the BSD1 transcription factor in vegetative growth in tomato, we further generated *BSD1* null knockout mutant (termed *BSD1‐KO*) by employing the clustered regularly interspaced short palindromic repeats (CRISPR) – associated protein 9 (CRISPR‐Cas9) system. We used double‐guide RNAs (dgRNAs) targeting the first exon of *BSD1* to generate two homozygous mutant lines (*BSD1‐KO*) with a 1 and 8 bp deletion, resulting in frame shifts that generated premature stop codons after the 59th and 45th amino acid residues, respectively (Figure S1E, Supporting Information). The transgenic lines were advanced to T_1_ populations to identify individuals that contained homozygous deletion but the Cas9 cassette segregated out. T_2_ null *BSD1‐KO* mutant populations were further generated and used for phenotypic analyses. Not to our surprise, *BSD1‐KO* plants displayed a typical dwarfism phenotype, with significantly reduced height compared to non‐transgenic wild type (Ailsa craig, AC) tomato plants (**Figure** **1**A). At six‐week‐old age, the average height of *BSD1‐KO* plants was shorter than that of AC plants (Figure 1B). We further measured the node number and average length of internodes and found the average internode length of *BSD1‐KO* plants was significantly shorter than that of AC plants (Figure 1D) Significantly, there was no significant difference of the internode numbers between *BSD1‐KO* and AC plants (Figure 1C). To determine the cellular basis of *BSD1‐KO* dwarf phenotype, particularly whether the abnormal internode elongation was caused by alterations in cell number or cell size, longitudinal sections of internodes were examined. As shown in Figure 1E, in the longitudinal section, cells were significantly smaller and tightly stacked in *BSD1‐KO* internodes compared to those in AC internodes. Thus, shortened internodes caused by altered cell expansion appear to count for the dwarf phenotype of *BSD1‐KO* plants (Figure 1E,F). Taken together, these data suggest that BSD1 controls vegetative growth in tomato via regulation of cell expansion‐based internode elongation.

### 2.2 BSD1 Interacts with SINA Ubiquitin Ligases

**Figure 2:**
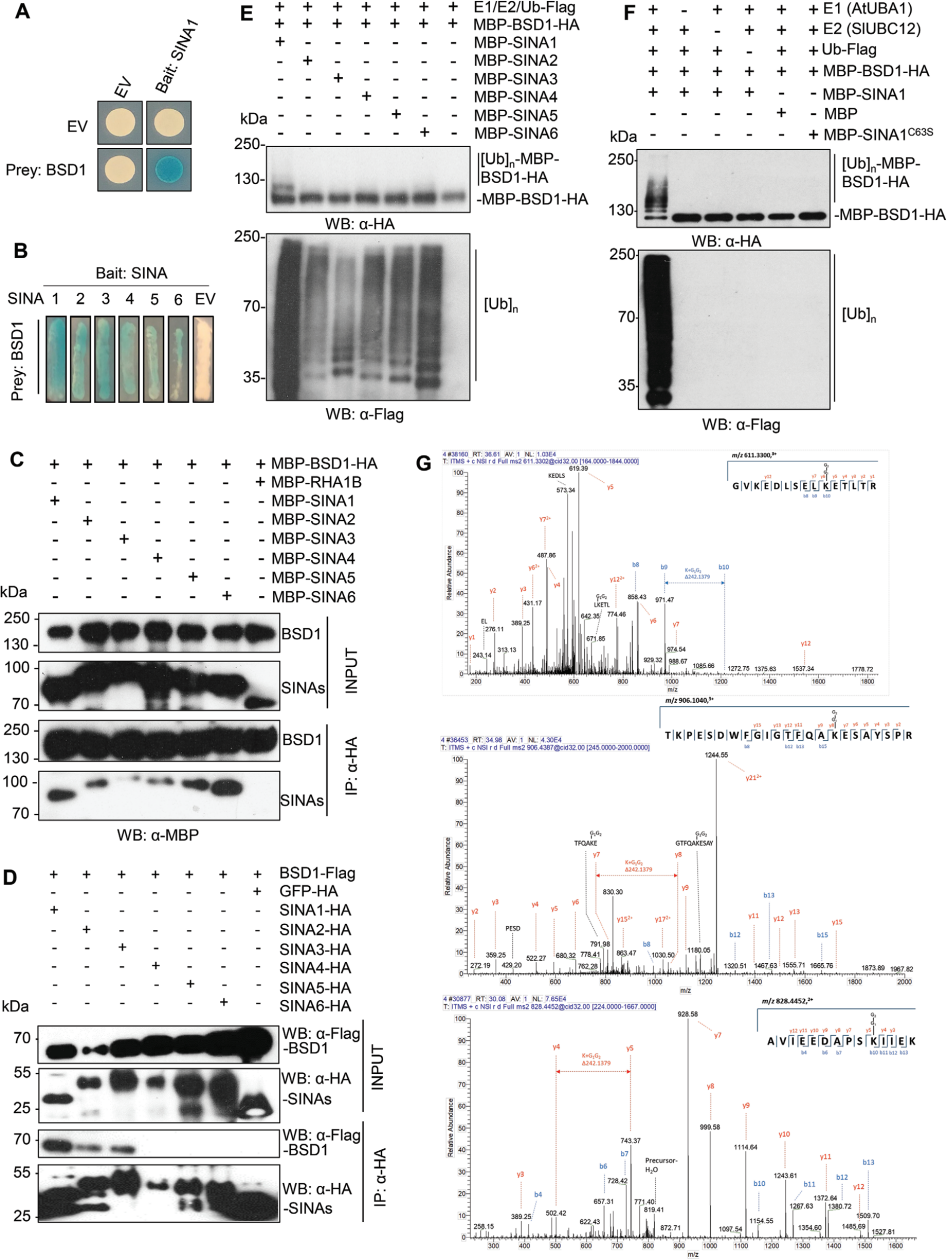
SINA1 interacts with and ubiquitinates BSD1 at lysine residues K93, K293 and K362. A,B) Y2H assay showing BSD1 interacts with SINAs, as indicated by blue coloration of yeast cells (transformed with the bait pEG202 constructs, prey pJG4‐5 constructs, or control empty vector (EV)) grown on the selective medium containing X‐Gal. C) In vitro binding assay showing that BSD1 directly interacts with SINAs. Epitope‐tagged recombinant protein MBP‐BSD1‐HA was incubated with six MBP‐SINAs or unrelated MBP‐RHA1B protein and was captured by anti‐HA affinity beads, followed by Western blotting using anti‐MBP antibody. D) co‐IP assay indicating BSD1 interacts with SINA1/2/3 in plant cells. Appropriate combinations of *in planta* expression constructs were co‐expressed in *N. benthamiana* leaves as indicated together with 50 µm MG132 for two days. Protein extracts were immunoprecipitated by anti‐HA affinity matrix and analyzed by Western blotting using appropriate antibodies. BSD1‐Flag was specifically co‐immunoprecipitated with SINA1/2/3‐HA. E) In vitro ubiquitination of BSD1 by SINA1. In vitro ubiquitination assays were conducted in the presence of ATP with the indicated combinations of proteins. The smear banding pattern recognized by anti‐HA antibody indicated BSD1 is specifically ubiquitinated by SINA1 (lane 1, top panel). Anti‐Flag indicated total ubiquitination catalyzed by six SINAs (bottom panel). F) SINA1 ubiquitinates BSD1 in an E3‐dependent manner. MBP‐SINA1^C63S^ (mutant without ubiquitin ligase activity) was included for the ubiquitination assay (line 6), only the BSD1 in the presence of recombinant E1, E2, MBP‐SINA1 and FLAG‐Ub showed polyubiquitination (lane 1, top panel). Anti‐Flag indicated total ubiquitination mediated by SINA1 (line 1, bottom panel). G) SINA1 ubiquitinates BSD1 at residues K93, K293 and K362. Tandem mass spectrometric analysis of ubiquitinated peptides from the BSD1 protein was conducted. Three key peptides were identified: A triply charged peptide at m/z 611.3300 corresponding to the sequence GVKEDLSELKETLTR with a ubiquitin remnant (GlyGly) modification at lysine residue 93 (K93) (upper panel); A triply charged peptide at m/z 906.1040 corresponding to the sequence TKPESDWFGIGTFQAKESAYSPR with a ubiquitin remnant modification at K293 (middle panel); A doubly charged peptide at m/z 828.4452 corresponding to the sequence AVIEEDAPSKIIEK with a ubiquitin remnant modification at K362 (lower panel). The fragmentation ions were annotated on the peptide sequence: b ions labeled in blue and y ions in red. All experiments have been repeated at least twice with similar results.

Next, we sought to investigate the regulation of the BSD1 transcription factor at the protein level. Coincidently, during our previous study on the substrates of SINA ubiquitin ligases,^[^ ^22^ ^]^ we identified BSD1 as a putative interactor of SINA1 in yeast two‐hybrid (Y2H) screening assay (**Figure** **2**A). Tomato contains six SINA proteins (SINA1‐6) that possess RING‐dependent ubiquitin ligase activity, despite their functionality has not been well studied.^[^ ^22^ ^]^ This gave rise to a hypothesis that BSD1 may be a substrate of and regulated by SINA ubiquitin ligases. To verify this hypothesis, we first verified the interaction of BSD1 with six SINA proteins by Y2H assay (Figure 2B). Next, we determined whether BSD1 interacts with SINA1‐6 in vitro and in plant cells. In vitro binding assay was carried out by incubating the epitope‐tagged recombinant protein MBP‐BSD1‐HA with recombinant MBP‐SINA1‐6 or the control MBP‐RHA1B protein (an unrelated protein from nematode *Globodera pallida*).^[^ ^27^ ^]^ As shown in Figure 2C, anti‐HA antibody pulled down MBP‐BSD1‐HA together with MBP‐SINA1‐6, but not with the control MBP‐RHA1B, revealing the specific binding of BSD1 to SINA1‐6. Since the in vitro protein interaction takes place in an artificial condition and may not reflect the interaction in plant cells, *in planta* association of BSD1 with SINAs was examined by co‐immunoprecipitation (co‐IP) assay. Epitope‐tagged BSD1 (*BSD1‐Flag*) was co‐expressed with epitope‐tagged SINAs (*SINA1‐6‐HA*) or GFP (*GFP‐HA*) in *Nicotiana benthamiana* leaves via *Agrobacterium*‐mediated transient expression. It is notable that SINAs, as functional ubiquitin ligase, may trigger degradation of BSD1 *in planta*, thus the proteasome‐specific inhibitor MG132 was included in the agrobacterial inoculum. After protein extraction and immunoprecipitation with the anti‐HA antibody matrix, the immunoprecipitated protein complex was subjected to Western blotting using the anti‐FLAG antibody. As shown in Figure 2D, the BSD1‐Flag protein was detected in the anti‐HA antibody‐immunoprecipitated complex from the leaf tissue expressing *BSD1‐Flag* with *SINA1/2/3‐HA*, but not in the immunoprecipitated complex from the leaf tissue expressing *BSD1‐Flag* with *SINA4/5/6‐HA* or *GFP‐HA*, suggesting BSD1 specifically interacts with SINA1/2/3 in plant cells.

### 2.3 SINA1 Targets BSD1 for Ubiquitination at K93, K293, and K362

Given that the ubiquitin ligases SINA1/2/3 specifically interact with BSD1 both in vitro and in vivo (Figure 2A–D), we determined whether BSD1 is the enzymatic substrate of and can be directly ubiquitinated by SINA proteins. In vitro ubiquitination of the recombinant BSD1 protein (MBP‐BSD1‐HA) mediated by six recombinant SINAs (MBP‐SINA1‐6) was examined in the presence of recombinant E1 (AtUBA1), E2 (SlUBC12) and ubiquitin (Flag‐Ub). As shown in Figure 2E, despite all six SINAs showed ligase activity (Figure 2E, bottom panel), only SINA1 trans‐ubiquitinated BSD1, resulting in a smear pattern of BSD1 protein, which was detected by Western blotting using the anti‐HA antibody (Figure 2E, top panel, lane 1). To further verify the SINA1‐mediated ubiquitination of BSD1 is dependent on the ligase activity, the ubiquitin ligase‐deficient mutant SINA1^C63S^ was included in the in vitro ubiquitination assay.^[^ ^25^ ^]^ The result indicated that ubiquitination did not occur in control reactions in which either one of the necessary components was missing or MBP or the ubiquitin ligase‐deficient mutant SINA1^C63S^ was used (Figure 2F, top and bottom panels, lanes 2–6). Thus, we conclude that SINA1 specifically ubiquitinates BSD1 in an E3‐dependent manner.

To identify the possible ubiquitination sites in BSD1, an in vitro ubiquitination of BSD1 by SINA1 was conducted and proteins were separated by SDS‐PAGE, the resulting gel band containing poly‐ubiquitinated BSD1 was excised, and in‐gel digested by trypsin. Using liquid chromatography‐tandem mass spectrometry (LC‐MS/MS), we identified three key peptides: A triply charged peptide at m/z 611.3300 corresponding to the sequence GVKEDLSELKETLTR with a ubiquitin remnant (GlyGly) modification at lysine residue 93 (K93) (Figure 2G, upper panel); A triply charged peptide at m/z 906.1040 corresponding to the sequence TKPESDWFGIGTFQAKESAYSPR with a ubiquitin remnant modification at K293 (Figure 2G, middle panel); A doubly charged peptide at m/z 828.4452 corresponding to the sequence AVIEEDAPSKIIEK with a ubiquitin remnant modification at K362 (Figure 2G, lower panel). These findings indicated that SINA1 ubiquitinates BSD1 at three specific lysine residues: K93, K293, and K362. To further verify the ubiquitination of these three specific lysine residues by SINA1, we mutagenized the three lysine to arginine residues (K93R, K293R, and K362R) and determined whether this would compromise BSD1 ubiquitination by SINA1. Interestingly, our in vitro ubiquitination assay indicated only marginal decrease of SINA1‐mediated ubiquitination of BSD1^K93R/K293R/K362R^ mutant compared to that of the WT BSD1 protein (Figure S2C, Supporting Information), suggesting other lysine residues can still be ubiquitinated by SINA1 when these three lysine residues are blocked. Significantly, when we attempted to determine the effect of SINA1 on the accumulation of BSD1^K93R/K293R/K362R^ mutant in plant cells, we did not detect BSD1^K93R/K293R/K362R^ mutant protein by Western blotting (Figure S2E, Supporting Information), which was the same case for the single amino acid substitution mutants BSD1^K93R^, BSD1^K293R^ and BSD1^K362R^, despite all K‐to‐R mutant constructs were expressed well, as indicated by similar levels of their transcripts (Figure S2D, Supporting Information). Taken together, the results suggest that, when incubated in vitro, SINA1 can ubiquitinate BSD1 at least at three lysine sites K93, K293 and K362, which are also important for BSD1 accumulation in plant cells)

### 2.4 SINA1 Promotes Proteasome‐Mediated Degradation of BSD1

**Figure 3:**
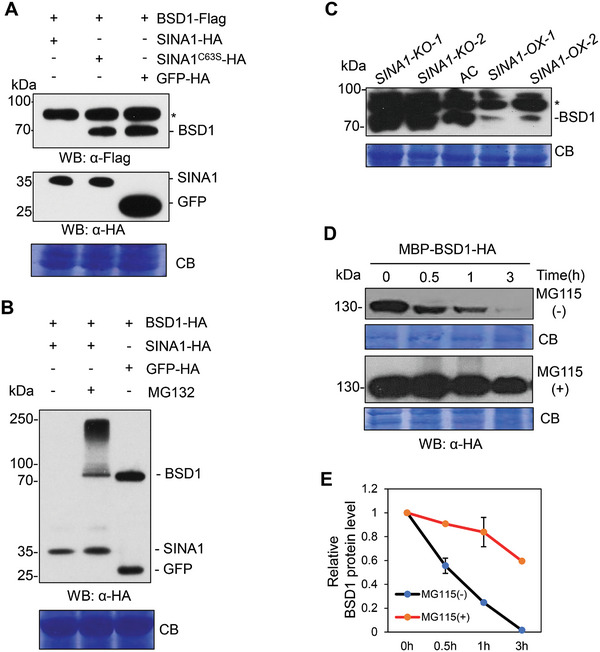
SINA1 promotes proteasome‐mediated degradation of BSD1. A) BSD1 degradation promoted by SINA1 requires its ubiquitin ligase activity. SINA1, but not the ligase‐deficient SINA1^C63S^ mutant or GFP, triggered degradation of BSD1 when co‐expressed in *N. benthamiana* leaves. The asterisk indicates nonspecific bands cross‐reacting with anti‐Flag antibody. Coomassie blue staining (CB) verifies equal loading of proteins. B) SINA1 promotes proteasome‐mediated degradation of BSD1. Epitope‐tagged BSD1‐HA was co‐expressed with SINA1‐HA or GFP‐HA in *N. benthamiana* protoplasts with or without presence of 50 µm proteasome inhibitor MG132. SINA1‐triggered degradation of BSD1 was abolished in the presence of MG132 and a smear banding pattern of BSD1 protein was detected, which likely represents the poly‐ubiquitinated form of BSD1. Coomassie blue staining (CB) verifies equal loading of proteins. C) SINA1 promotes the degradation of BSD1 in tomato. BSD1 protein levels in *SINA1‐OX, SINA1‐KO*, and AC were detected using anti‐BSD1 antibody. Coomassie blue staining (CB) verifies equal loading of proteins. D) Proteasome inhibitor prevents the degradation of BSD1. Cell lysates from one‐month‐old *SINA1‐OX* plants were incubated with recombinant MBP‐BSD1‐HA protein with or without 50 µm proteasome‐specific inhibitor MG115. The protein levels of BSD1 were detected using anti‐HA antibody at different time points after incubation. Coomassie blue staining (CB) verifies equal loading of proteins. E) Quantification of BSD1 signal shown in (j) using Image J software. The BSD1 protein levels at 0 h were set as 1. Data are means ± SD. All experiments have been repeated at least twice with similar results.

Next, we determined whether SINA1‐mediated ubiquitination of BSD1 results in proteasome‐dependent degradation in plant cells. To this end, we co‐expressed epitope‐tagged *BSD1‐Flag* with *SINA1‐HA* or *GFP‐HA* in *N. benthamiana* leaves and monitored the BSD1‐Flag protein levels. The *in planta* expression assay indicated that SINA1, but not GFP, triggers BSD1 degradation in plant cells (**Figure** **3**A, lane 3). Significantly, this degradation was dependent on the ubiquitin ligase activity of SINA1 since BSD1‐Flag protein accumulated normally when co‐expressed with the ubiquitin ligase‐deficient *SINA1^C63S^* mutant (Figure 3A, lane 2). To further determine whether SINA1‐promoted BSD1 destruction is mediated by proteasome degradation, we adopted the *N. benthamiana* protoplast system to co‐express *BSD1* and *SINA1* with or without the presence of the proteasome‐specific inhibitor MG132. As expected, co‐expression of *BSD1* with *SINA1*, but not *GFP*, resulted in no accumulation of BSD1 in protoplasts (Figure 3B, lane 1). However, application of MG132 dramatically abolished the degradation of BSD1 by SINA1 and, significantly, a smear banding pattern of BSD1 protein was detected as well, which likely represented the poly‐ubiquitinated form of BSD1 (Figure 3B, lane 2). Taken together, our data suggest that SINA1 directly ubiquitinates BSD1 for the proteasome‐mediated degradation.

To further determine the regulation of BSD1 by SINA1 in tomato in the context of BSD1 accumulation, we generated transgenic tomato plants with overexpression (termed *SINA1‐OX*) or knockout (termed *SINA1‐KO*) of *SINA1* gene, among which the *SINA1‐OX* plants over‐expressed the *SINA1* transgene under the control of a cauliflower mosaic virus (CaMV) 35S promoter and *SINA1‐KO* plants were created via a CRISPR‐Cas9 gene editing system targeting the second exon of *SINA1*, respectively. More than 10 *SINA1‐OX* overexpressing lines were generated and two of them with the highest expression levels were advanced to T_2_ generation for further analysis (Figure S2A, Supporting Information). In the meantime, four different guide RNAs were used to target the second exon of *SINA1*, 14 individual *SINA1‐KO* mutant lines were generated, 12 of them had an A insertion and the other two lines harbored a 5‐bp deletion at the second exon of *SINA1* gene, resulting in a frame shift with a premature stop codon after the 193rd and 167th amino acid residues, respectively (Figure S2B, Supporting Information). The *SINA1‐KO* lines were advanced to T_2_ populations to obtain null *SINA1‐KO* mutant populations without the Cas9 cassette and used for further phenotypic analysis. We then examined the endogenous BSD1 protein levels in *SINA1‐OX*, *SINA1‐KO*, and AC tomato plants by Western blotting using a custom anti‐BSD1 antibody. As shown in Figure 3C, BSD1 was degraded in *SINA1‐OX* plants compared to AC and *SINA1‐KO* plants. This indicates that overexpression of *SINA1* triggers increased BSD1 degradation in tomato and suggests that SINA1 is a ubiquitin ligase regulating BSD1 accumulation. To further verify SINA1 regulates BSD1 through the ubiquitin‐proteasome pathway in tomato, recombinant protein MBP‐BSD1‐HA was incubated with cell lysates extracted from *SINA1‐OX* transgenic plants with or without the addition of the proteasome inhibitor MG115. As shown in Figure 3D, the SINA1‐mediated degradation of BSD1 was remarkably abolished in the presence of MG115. Thus, based on our molecular and genetic data, we conclude that SINA1 targets BSD1 for ubiquitin‐mediated proteasomal degradation, thereby regulating its protein levels in tomato.

### 2.5 SINA1 Functions through BSD1 to Control Vegetative Growth in Tomato

**Figure 4:**
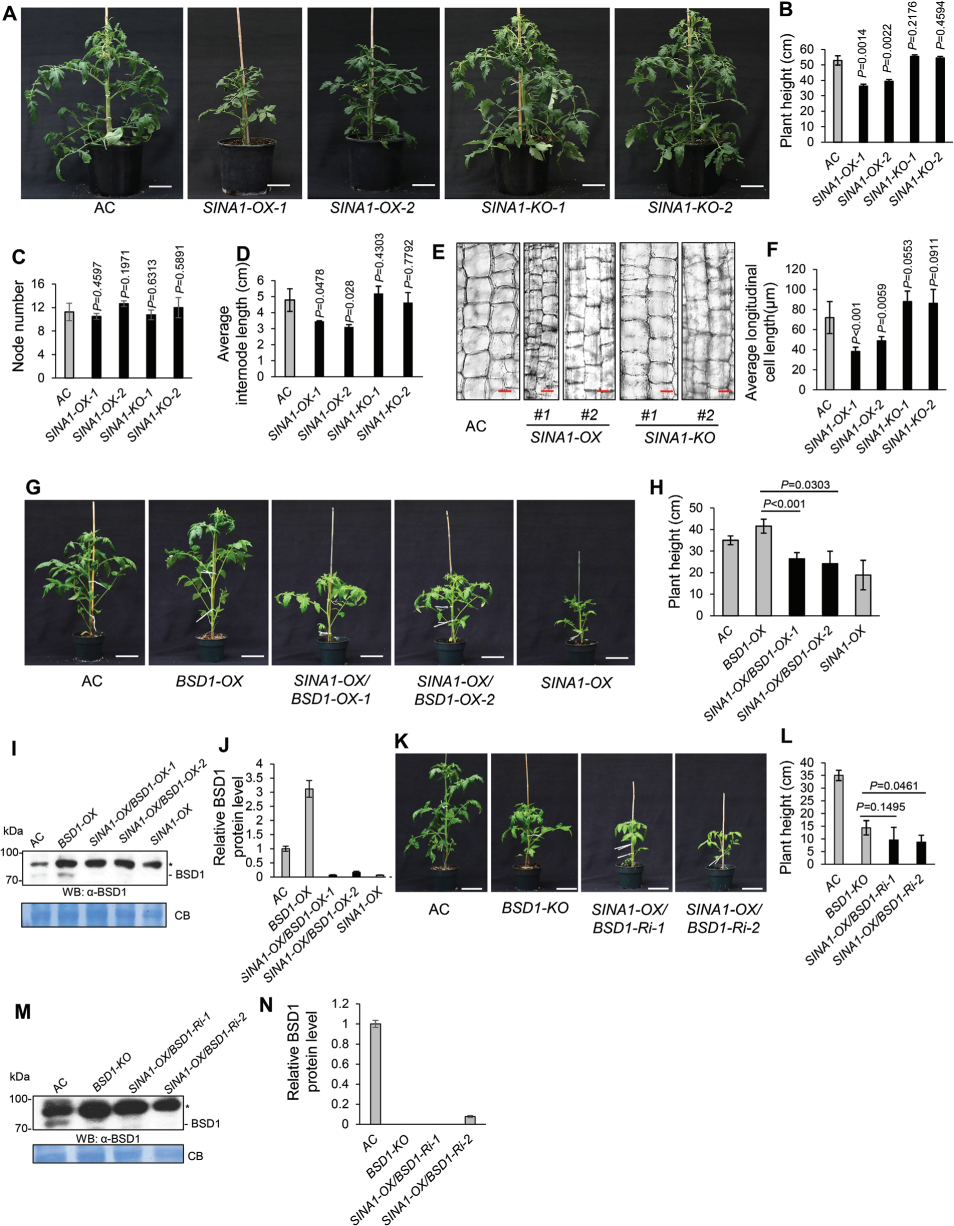
SINA1 functions through BSD1 to control vegetative growth. A) The morphologic appearance of representative six‐week‐old AC, *SINA1‐OX*, and *SINA1‐KO* plants. White scale bar, 10 cm. B‐D) Statistical analyses on plant height B), node number C), and average internode length D) of plants in (A). N = 4. *P* values were indicated in the graph. E) Longitudinal section and staining with toluidine blue for the 3^rd^ internode stems of two‐month‐old AC, *SINA1‐OX* and *SINA1‐KO* plants. Scale bar, 50 µm. F) Quantification of longitudinal length of cells in (E). N = 3. *P* values are indicated in the graph. G‐N) SINA1 genetically regulates BSD1 in the context of vegetative growth. Plants in (G) and (K) represent the morphologic appearance of representative five‐week‐old AC, *BSD1‐OX, SINA1‐OX/BSD1‐OX*, *SINA1‐OX, BSD1‐KO, SINA1‐OX/BSD1‐Ri* plants. Scale bar, 10 cm. Quantifications of the plant height of plants in (G) and (K) are presented in (H) and (L), respectively. N = 4. *P* values are indicated in the graph. Western blotting analyses using customized anti‐BSD1 antibody indicate overexpression of SINA1 triggers degradation of BSD1 in *SINA1‐OX/BSD1‐OX* (I, J) and *SINA1‐OX/BSD1‐Ri* (M, N) plants. N = 3. *P* values are indicated in the graph. Data are means ± SD; ^*^ *p* < 0.05, two‐tailed *t*‐tests. All experiments have been repeated at least twice with similar results.

Given the fact that SINA1 ubiquitinates BSD1 for degradation and BSD1 positively regulates vegetative growth, it is likely that SINA1 may control vegetative growth via regulation of BSD1 protein levels in tomato. Thus, we grew *SINA1‐OX*, *SINA1‐KO* and AC tomato plants under the same growth conditions for phenotypic verification. As shown in **Figure** **4**A, *SINA1‐OX* plants exhibited a similar dwarfism phenotype as *BSD1‐KO* plants did, whereas *SINA1‐KO* were indistinguishable from AC plants. Significantly, statistical analysis indicated that the dwarf phenotype of *SINA1‐OX* plants was attributed to the shortened internodes (Figure 4B–D). Moreover, again resembling the causal cellular basis for the dwarfism of *BSD1‐KO* plants, *SINA1‐OX* internodes exhibited significantly shorter longitudinal cell length than AC internodes, whereas there was no difference in cell sizes between AC internodes and *SINA1‐KO* internodes (Figure 4E,F). These results suggest that SINA1 controls tomato vegetative growth, likely via direct regulation of the BSD1 transcription factor that is involved in regulation of cell elongation‐based internode growth. To further test this notion, we explored the genetic interaction between SINA1 and BSD1 in the context of regulation of vegetative growth. We conducted secondary transformation to generate two “double” transgenic tomato lines with both *SINA1* and *BSD1* genes being manipulated. The first one was generated by overexpression of *SINA1* in the background of *BSD1‐OX* plants. The second one was generated by overexpression of *SINA1* in the background of the non‐null *BSD1‐Ri* line, in which the *BSD1* gene expression was repressed ≈70%.^[^ ^26^ ^]^ Since SINA1 targets BSD1 for ubiquitination and degradation, it was logical to speculate that SINA1 acts dominantly over BSD1 in controlling vegetative growth and we anticipated 1.) Overexpression of *SINA1* in *BSD1‐OX* background should overpower the potential growth‐promotion capacity of *BSD1* overexpression, thereby resulting in a dwarfism phenotype; 2.) In contrast, overexpression of *SINA1* in *BSD1‐Ri* background should reinforce the consequence of *BSD1* knockdown, leading to enhanced dwarfism. As shown in Figure 4G, despite *BSD1‐OX* plants were slightly taller than the AC plants, introduction of the *SINA1* overexpression construct into *BSD1‐OX* plant conferred the resulting “double” transgenic *SINA1‐OX*/*BSD1‐OX* plant a dwarfism phenotype, which is consistent with the observation of barely detected BSD1 protein in the *SINA1‐OX*/*BSD1‐OX* plant (Figure 4I,J); On the other hand, when *SINA1* was overexpressed in the *BSD1‐Ri* background plant, the dwarfism of resulting *SINA1‐OX*/*BSD1‐Ri* “double” transgenic plant was comparable to that of the *BSD1‐KO* plant (Figure 4K) and, again, BSD1 protein accumulation was dramatically reduced in the *SINA1‐OX*/*BSD1‐Ri* “double” transgenic plant due to overexpression of *SINA1* (Figure 4M,N). Thus, these data provide genetic evidence supporting the notion that SINA1 functions through BSD1 to control vegetative growth in tomato.

### 2.6 BSD1 activates the BRG1 Gene through Binding to a Binding Motif in Its Promoter

**Figure 5:**
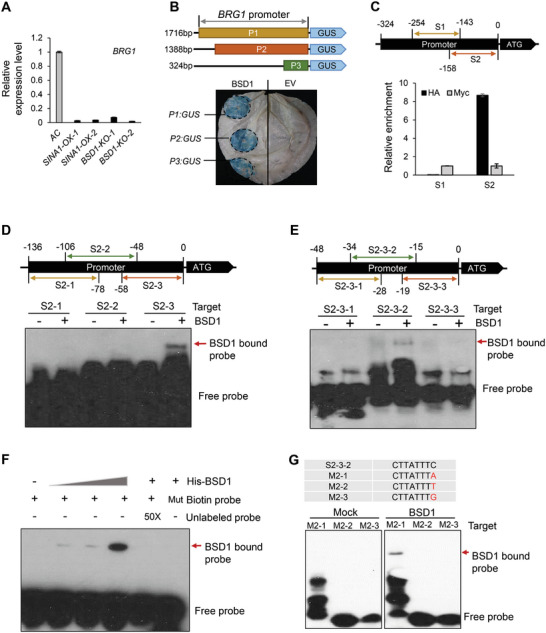
BSD1 regulates *BRG1* via directly binding to its promoter. A) The relative expression levels of *BRG1* in AC, *SINA1‐OX*, and *BSD1‐KO* plants. B) Identification of a minimal promoter region of *BRG1* activated by BSD1. P1, P2, and P3 represent *BRG1* promoter regions upstream of start codon ATG as indicated (top panel). The GUS reporter genes were co‐expressed with BSD1 or empty vector control (EV) in *N. benthamiana* leaves and histochemical GUS staining was conducted to determine the potential promoter activated by BSD1 (Bottom panel). C) ChIP‐PCR assay defines the promoter region of *BRG1* associated with BSD1 protein. The relative enrichment was determined using the quantitative data from real‐time PCR. Anti‐HA or ‐Myc (served as a negative control) beads were used to pull down BSD1‐HA protein from internodes of transgenic *BSD1‐OX* plants. DNA recovered from the immunoprecipitated complex was applied for ChIP‐qPCR analysis. D, E) Narrowing down the BSD1 binding fragments located in the *BRG1* promoter. A serial biotin‐labeled DNA fragment truncations (from S2‐1/2/3 to S2‐3‐1/2/3 as indicated in D and E, respectively) were used as probes for the EMSA assay with or without the presence of BSD1 recombinant protein. F) EMSA competition assay. BSD1 recombinant protein was incubated with biotin‐labeled S2‐3‐2 fragment as a probe against 50‐fold more unlabeled S2‐3‐2 fragment or biotin‐labeled mutant fragment CTTAAAACTCCATTTTACTC. G) CTTATTTC/A is a BSD1‐binding motif. EMSA assay using biotin‐labeled single nucleotide mutant probes (M2‐1/2/3) indicates BSD1 recombinant protein specifically binds to CTTATTTC and CTTATTTA sequences. All experiments have been repeated at least twice with similar results.

Next, we sought to investigate tomato genes regulated by the SINA1‐BSD1 module in the context of vegetative growth, particular genes directly regulated by the BSD1 transcription factor. Previously we identified a large number of genes directly or indirectly regulated by BSD1 by gene profiling with *BSD1‐Ri* plants in comparison with AC plants.^[^ ^26^ ^]^ To further determine the potential effect of SINA1 on expression of these genes, we conducted similar RNA‐seq analysis on *SINA1‐OX* plants in comparison with AC plants (Figure S3A,B, Supporting Information) and found 35 genes were down‐regulated in both *BSD1‐Ri* and *SINA1‐OX* plants compared to AC plants with more than two folds change (Figure S3C, Supporting Information). To identify the direct target genes of the SINA1‐BSD1 regulatory module, four genes of the ones with the highest expression change were selected and their promoters were PCR‐amplified and fused with a *β‐glucuronidase* (*GUS*) gene (Figure S3D,E, Supporting Information). The resulting *GUS* reporter genes were transiently co‐expressed with *BSD1* or empty vector in *N. benthamiana* leaves, and the GUS activities were monitored by histochemical GUS staining. Strong GUS activity was observed when the *GUS* reporter gene driven by the promoter of *Solyc07g054310* (termed *BSD1‐regulated gene 1* (*BRG1*)) was co‐expressed with *BSD1* in *N. benthamiana* leaves (Figure S3E, Supporting Information), suggesting *BRG1* promoter was activated by the BSD1 transcription factor. Consistent with this promoter analysis, the *BRG1* gene was significantly down‐regulated in *SINA1‐OX* and *BSD1‐KO* plants (**Figure** **5**A), implying BSD1 regulates *BRG1* expression in tomato. To narrow down the minimal promoter region, two truncated fragments (termed P2, 3) of *BRG1* promoter were tested and the region of 324 bp (P3) upstream the start codon could act as a functional promoter activated by BSD1 (Figure 5B). The binding of BSD1 to this P3 (−324 to 0 bp) region was also examined by a ChIP‐PCR assay using *BSD1‐OX* transgenic plants, which pointed out the segment of −158 to 0 bp (termed S2) upstream start codon acts as a putative binding site of BSD1 (Figure 5C).

Since BSD1 was identified as a novel plant‐specific transcription factor and its binding sites were unknown, it was necessary to define core binding sites of BSD1 for mining its potential functions. To this end, based on the results of *GUS* reporter gene and ChIP‐PCR assays, we adapted electrophoresis mobility shift assay (EMSA) to determine the exact binding sequences of BSD1. First, three overlapped oligonucleotide probes (termed S2‐1/2/3, −136 to −78 bp, −106 to −48 bp, and −58 to 0 bp upstream ATG, respectively) were examined for the capability of binding to the epitope‐tagged recombinant BSD1 protein (His‐BSD1). The EMSA assay indicated the core element is located at the −48 to 0 bp region (Figure 5D; Figures S2 and S3, Supporting Information). Three further shortened oligonucleotide probes (termed S2‐3‐1/2/3) were examined and the S2‐3‐2 probe (5′‐CTTATTTCTCCATTTTACTC‐3′) bound to BSD1 (Figure 5E). This EMSA result was also confirmed by competition and binding specificity assays, in which it was found that the binding of BSD1 to S2‐3‐2 probe is abolished by addition of 50‐fold unlabeled S2‐3‐2 probe and BSD1 does not bind to the S2‐3‐2 mutant probe containing a single nucleotide mutation (Figure 5F). At last, serial truncations of S2‐3‐2 oligonucleotides with single nucleotide deletion or mutation were examined and the EMSA results indicated that minimal 5′‐CTTATTTC/A‐3′ oligonucleotide (termed as BBS motif, standing for BSD1 binding site) was sufficient for binding to BSD1 (Figure 5G; Figure S4, Supporting Information). Taken together, our results suggest that the BSD1 transcription factor directly activates the *BRG1* gene, presumably via binding to the BBS motif in the promoter of *BRG1* gene.

### 2.7 BRG1 Encodes a Hypothetic Protein Involved in Vegetative Growth in Tomato

**Figure 6:**
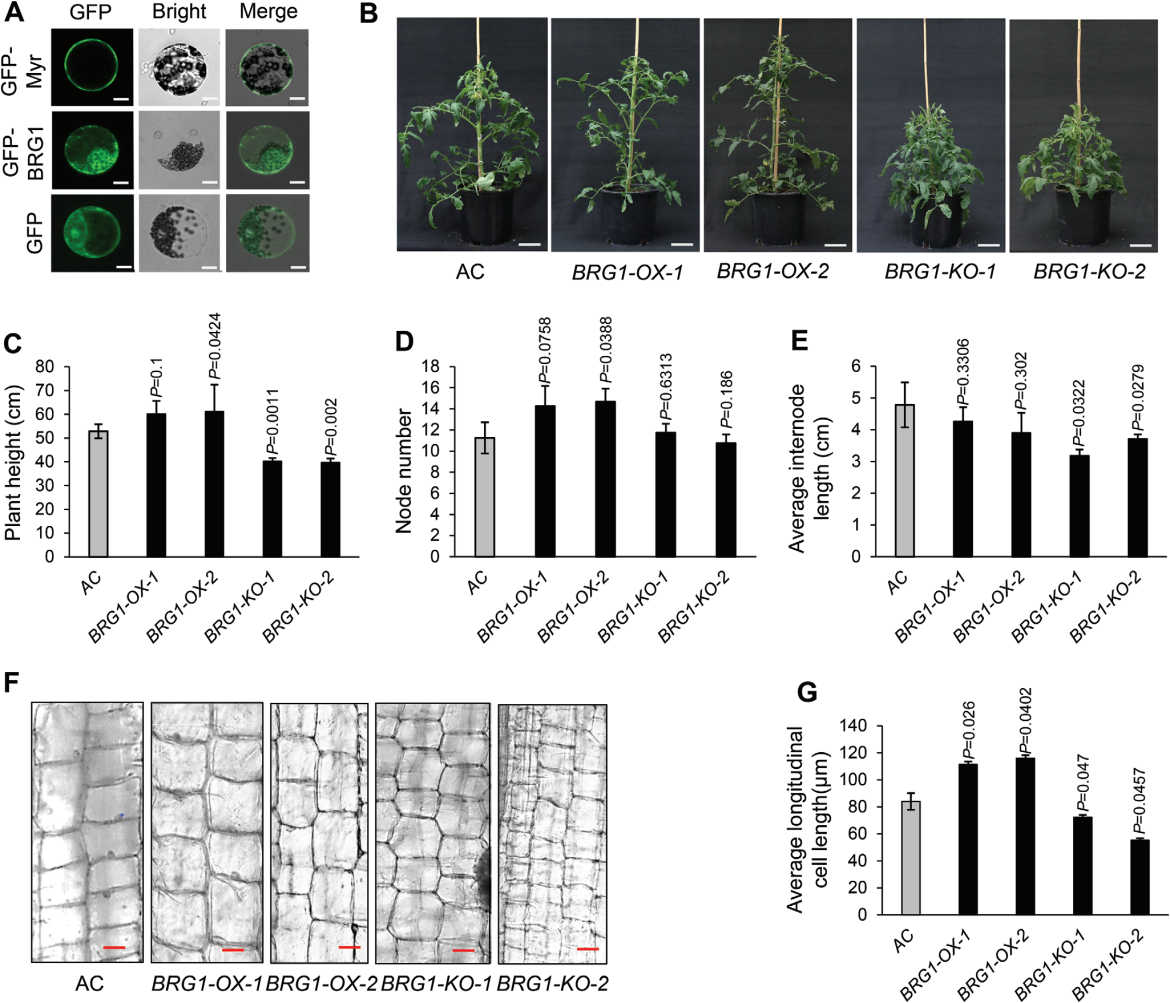
BRG1 is involved in vegetative growth in tomato. A) Subcellular localization of the BRG1 protein. CaMV 35S promoter‐driven GFP‐Myr (a known plasma membrane‐localized protein), GFP or GFP‐BRG1 constructs were transiently expressed in *N. benthamiana* protoplasts for 36 h, followed by fluorescent microscopy to detect fluorescence signal‐derived from GFP‐Myr, GFP or GFP‐BRG1. Scale bar, 20 µm. B) The morphologic appearance of representative six‐week‐old AC, *BRG1‐OX*, and *BRG1‐KO* plants. Scale bar, 10 cm. C–E) Quantification of the plant height C), node number (D), and average internode length (E) of plants in (B). N = 4. *P v*alues are indicated in the graph. F) Longitudinal section and staining with toluidine blue for the 3^rd^ internode stem of two‐month‐old AC, *BRG1‐OX*, and *BRG1‐KO* plants. Scale bar, 50 µm. G) Quantification of longitudinal length of cells in (F). N = 3. *P* values are indicated in the graph. Data are means ± SD; ^*^ *p* < 0.05, two‐tailed *t*‐tests. All experiments have been repeated at least twice with similar results.

Sequence analysis predicted that *BRG1* encodes a hypothetic protein of 127 amino acids without known functions. Transmembrane prediction conducted by TMHMM‐2.0 (TMHMM – 2.0 – Services – DTU Health Tech) indicated that BRG1 possesses a terminal putative transmembrane (TM) motif (Figure S5A, Supporting Information). To determine the subcellular localization of BRG1 in plant cells, GFP, GFP‐tagged Myr (GFP‐Myr, a known plasma membrane‐localized protein ^[^ ^28^ ^]^) or BRG1 (GFP‐BRG1) driven by the CaMV 35S promoter were expressed in *N. benthamiana* protoplasts for fluorescent microscopy assay. The result suggests the BRG1 protein is localized in plasma membrane and cytoplasm (**Figure** **6**A).

Since *BRG1* is a direct target gene regulated by BSD1 that is a positive regulator of vegetative growth, it is necessary to determine whether *BRG1* plays a role in vegetative growth in tomato. To this end, we generated and characterized *BRG1‐*overexpression (termed *BRG1‐OX*) and null *BRG1* knockout (termed *BRG1‐KO*) lines, in which *BRG1‐OX* plants overexpressed the *BRG1* gene under the control of the strong CaMV 35S promoter and *BRG1‐KO* plants were generated using the CRISPR‐Cas9 gene editing system targeting the N‐terminal *BRG1*, respectively. We generated more than seven *BRG1‐OX* lines and the two lines exhibiting the highest expression levels of *BRG1* were advanced to T_2_ homozygous populations for further analysis (Figure S5B, Supporting Information). In the meantime, multiple homozygous *BRG1‐KO* lines were generated and three individual lines containing a 1, 2 bp deletion, and a 1 bp insertion in *BRG1* gene, which resulted in frame shifts with premature stop codons after the 56th, 63rd, and 64th amino acid residue, respectively, were selected for further phenotypic analysis (Figure S5C, Supporting Information). The transgenic lines were advanced to T_1_ populations to identify individuals that contained homozygous deletion but the Cas9 cassette segregated out. T_2_ null *BRG1‐KO* mutant populations were further generated for phenotypic analysis. Not surprisingly, *BRG1‐KO* plants exhibited a dwarfism phenotype (Figure 6B) that was attributed to the significantly reduced plant height caused by shortened internode length (Figure 6C–E), which resembles the phenotypes of *BSD1‐KO* and *SINA1‐OX* plants. In contrast, *BRG1‐OX* plants were slightly taller and had more internodes than AC plants (Figure 6C,D). Since the function of BRG1 protein is unknown, particularly its role in development and growth awaits investigation, we sought to determine whether it plays a role in regulation of cell expansion that contributes to growth of the internode. To achieve this, longitudinal sections of internodes of *BRG1‐OX*, *BRG1‐KO*, and AC plants were examined. As shown in Figure 6F, the longitudinal cell length of *BRG1‐KO* internodes was smaller, but the longitudinal cell length of *BRG1‐OX* internodes was greater than that of AC internodes, suggesting BRG1 plays a positive role in regulation of cell expansion in internode tissues.

### 2.8 The SINA1‐BSD1 Module Regulates the Expression of GA Biosynthesis Genes Promoting GA Production in Tomato

**Figure 7:**
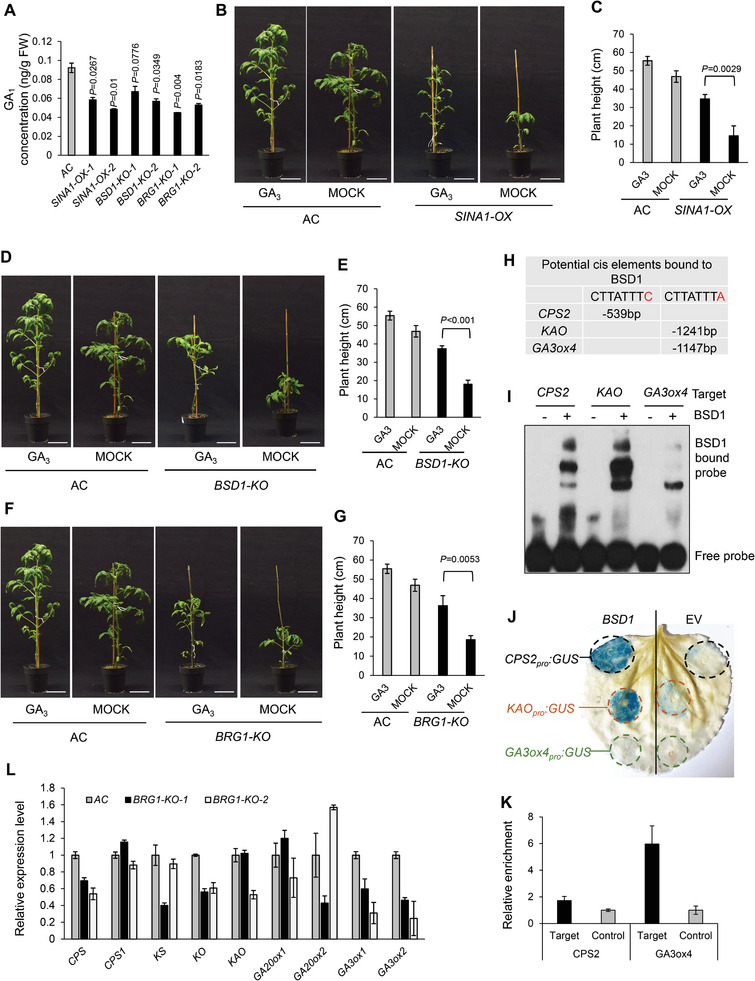
The SINA1‐BSD1 module regulates vegetative growth involving GA biosynthesis. A) Quantification of GA_1_ contents in the internode stems of AC, *SINA1‐OX*, *BSD1‐KO*, and *BRG1‐KO* transgenic plants. B, D, F) Exogenous application of bioactive GA_3_ restores the vegetative growth of *SINA1‐OX* B), *BSD1‐KO* D), and *BRG1‐KO* F) transgenic plants. Five‐week‐old plants were sprayed with 100 µm GA_3_ daily for one week. Scale bar, 10 cm. Note that the AC control images in (B, D, F) are identical and from the same experiment. C, E, G) Quantification of the plant height of *SINA1‐OX* (C), *BSD1‐KO* (E) and *BRG1‐KO* (G) transgenic plants. H) A BSD1‐binding motif is found in the promoter of three GA biosynthesis genes, *CPS2*, *KAO* and *GA3ox4*. I) EMSA assay indicates BSD1 directly binds to the promoter regions of GA biosynthesis genes *CPS2*, *KAO* and *GA3ox4*. J) BSD1 activates the promoters of *CPS2* and *KAO* genes. The GUS reporter genes, which are driven by promoter of *CPS2, KAO* or *GA3ox4*, were co‐expressed with *BSD1* or empty vector control (EV) in *N. benthamiana* leaves and histochemical GUS staining was conducted to determine the promoter activated by BSD1. K) BSD1 binds to the promoters of *CPS2* and *KAO* genes in vivo. Anti‐HA beads were used to pull down BSD1‐HA protein from internodes of AC (served as a negative control) and transgenic *BSD1‐OX* plants. DNA recovered from the immunoprecipitated complex was applied for ChIP‐qPCR analysis. L) Role of *BRG1* in the expression of GA biosynthesis genes. qPCR analyses on GA biosynthesis genes in the internodes of AC and *BRG1‐KO* transgenic plants indicate that four out nine GA biosynthesis genes are down‐regulated in *BRG1‐KO* plants, suggesting BRG1 plays a positive role in GA biosynthesis in tomato. Data are means ± SD; ^*^ *p* < 0.05, two‐tailed *t*‐tests. All experiments have been repeated at least twice with similar results.

It is well‐known that plant hormones, including auxin, BR and GA, promote vegetative growth in general. To determine the hormone pathway involved in SINA1‐BSD1‐mediated regulation of plant growth, we initially examined the expression of genes related to GA, BR and auxin hormones, including two GA‐related genes (*GA20ox1, GA3ox1*), two BR‐related genes (*CPD, CYP90B3*) and two auxin‐related genes (*FZY1, NIT*) in *SINA1‐OX* and *BSD1‐KO* plants (Figure S6G, Supporting Information). Our pilot qRT‐PCR assay on these genes revealed that only GA‐related genes (especially for *GA3ox1* gene) show consistent and significant expression changes in those plants (Figure S6G, Supporting Information). These results strongly suggested the SINA1‐BSD1 module primarily has influence on GA hormone‐mediated growth regulation, and, thus, we focused on GA hormone for further investigation. To test whether the SINA‐BSD1 module plays a role in GA biosynthesis, thereby regulating vegetative growth in tomato, we examined the GA contents in internodes of *SINA1‐OX, BSD1‐KO and BRG1‐KO* plants that exhibited dwarfism phenotype. The results indicated the bioactive GA_1_ contents in *SINA1‐OX, BSD1‐KO and BRG1‐KO* internodes were dramatically lower than that in the AC plants (**Figure** **7**A). Significantly, exogenous application of bioactive GA_3_ partially restored the vegetative growth of *SINA1‐OX, BSD1‐KO* and *BRG1‐KO* plants (Figure 7B–G; Figure S6A–F, Supporting Information), suggesting the dwarfism was likely due to GA deficiency in these transgenic plants. Next, it is logical to ask whether BSD1, as a transcription factor, directly regulates the expression of any GA biosynthesis genes involved in GA production. Since BSD1 specifically binds to the BBS motif to activate gene expression (Figure 5), we analyzed the promoter sequences of all 17 GA biosynthesis genes of the GA biosynthesis pathway and found three of them, *CPS2, KAO*, and *GA3ox4*, possess a BBS motif in their promoters (Figure 7H), suggesting the BSD1 transcription factor may directly regulate these GA biosynthesis genes. To verify this notion, we determined whether BSD1 directly binds to the promoter of *CPS2, KAO*, and *GA3ox4* genes by EMSA assay. The results indicated that the recombinant BSD1 protein is able to bind to the BBS motif‐containing promoter fragments of these genes at variable levels (Figure 7I). In addition, three *GUS* reporter genes (*CPS2_Pro_:GUS*, *KAO_Pro_:GUS* and *GA3ox4_Pro_:GUS*) driven by these promoters were transiently co‐expressed with *BSD1* or empty vector in *N. benthamiana* leaves, and the GUS activities were determined by histochemical GUS staining. Strong GUS activity was observed when *CPS2_Pro_:GUS* or *KAO_Pro_:GUS* was co‐expressed with *BSD1*, but not with the empty vector control, in *N. benthamiana* leaves (Figure 7J), suggesting *CPS2 and KAO* promoters were activated by the BSD1 transcription factor. It was notable that the *GA3ox4_Pro_:GUS* activity was very weak, implying BSD1 has less activation potential on *GA3ox4*, which could be due to weak binding of BSD1 with *GA3ox4* promoter (Figure 7I). To validate BSD1 binding to the BBS motif in vivo, we conducted ChIP assay and determined the binding of BSD1 to *CPS2* and *GA3ox4* promoters (Figure 7K). However, the promoter region of the *KAO* gene resists the qRT‐PCR‐based ChIP analysis due to long stretches of A and T nucleotides flanking the BBS motif (Figure S6L, Supporting Information). Nevertheless, these results suggest that BSD1 directly regulates at least three GA biosynthesis genes, *CPS2, KAO* and *GA3ox4*, promoting GA production to stimulate vegetative growth in tomato.

The relationship between BRG1 and GA biosynthesis was also investigated. Because there is no evidence showing BRG1 is a transcription factor, it is unlikely that BRG1 directly regulates GA biosynthesis genes. However, regardless of the functionality of BRG1, we determined the expression levels of nine GA biosynthesis genes in *BRG1‐KO* and AC plants by qPCR analysis. The results indicated that four of nine GA biosynthesis genes were down‐regulated in *BRG1‐KO* plants compared to the AC plants, suggesting that BRG1 may play a positive role in the GA biosynthesis genes despite that the molecular role of BRG1 in the context of gene expression is unknown (Figure 7L).

## 3 Discussion

In general, the levels of regulatory proteins, including transcription factors, are tightly regulated by the UPS, which is fulfilled via specific interaction between the substrate protein and the ubiquitin ligase prior to proteolysis in the proteasome.^[^ ^20^ ^]^ This is also the case with the tomato BSD1 transcription factor. The protein level of BSD1 is controlled by the ubiquitin ligase SINA1, which specifically interacts with and ubiquitinates BSD1, promoting the proteasome‐mediated degradation in plant cells (Figure 3). SINA1 belongs to the RING‐type Seven in absentia (SINA) ubiquitin ligase family that is conserved in both animals and plants. There are six SINA proteins (SINA1‐6) identified in tomato and they all possess ubiquitin ligase activity.^[^ ^22^ ^]^ Our previous gain‐of‐function analyses, conducted via transient expression of SINA proteins in the model plant *N. benthamiana* or overexpression of SINA proteins in transgenic tomato, have suggested SINA protein play important roles in diverse physiological processes in tomato, including that SINA2 regulates chlorophyll level in plant cells, SINA4 plays a positive role in defense signaling, and SINA5 is involved in flower development.^[^ ^22^ ^]^ In this study, SINA1 was found to negatively regulate vegetative growth by mediating the ubiquitination and degradation of a vegetative growth regulator BSD1. Significantly, although all six SINA proteins interacted with BSD1 in yeast and SINA1/2/3 interacted with BSD1 in plant cells (Figure 2), only SINA1 was able to ubiquitinate BSD1 promoting proteasome‐mediated degradation (Figures 2 and 3). This suggests that SINA1 is the main ubiquitin ligase responsible for BSD1 ubiquitination and degradation, which is further substantiated by the observation that the BSD1 protein was degraded in the *SINA1‐OX* plants (Figure 3C). However, functional redundancy possibly exists in SINA ubiquitin ligases, and other SINA proteins may also be involved in regulation of BSD1. Particularly, all six SINA proteins can be associated with each other for homo‐ or hetero‐dimerization,^[^ ^22^ ^]^ and the function of each SINA may be mutually affected by each other through interaction. For example, dimerization of SINA1 with other SINAs, particularly SINA2 and SINA3 that also interact with BSD1 in vivo, may facilitate SINA1‐mediated ubiquitination of BSD1. The genetic interaction between BSD1 and SINA1 was verified by the phenotypic analyses of the “double” *SINA1‐OX*/*BSD1‐OX* and *SINA1‐OX*/*BSD1‐Ri* transgenic tomato plants in comparison with the parental *BSD1‐OX* or *BSD1‐KO* transgenic tomato plants, respectively. Overexpression of *SINA1* subdued potential vegetative growth conferred by *BSD1* overexpression rendering a dwarfism phenotype with shortened‐internodes (Figure 4G,H) and enhanced the dwarfism of *BSD1‐Ri* plants caused by the incomplete suppression of *BSD1* gene (Figure 4K,L). Significantly, the dwarfism appears to be correlated with SINA1‐regulated BSD1 protein levels (Figure 4I,J,M,N), again suggesting SINA1 negatively regulates BSD1 in the context of controlling vegetative growth. However, given that SINA1 is localized in the nucleus and cytoplasm whereas BSD1 is exclusively localized in the nucleus,^[^ ^22^, ^26^ ^]^ it is possible that SINA1 may target and ubiquitinate other cytoplasmic proteins that may or may not be involved in vegetative growth.

Our study on in vitro SINA1‐mediated BSD1 ubiquitination sites highlights the complex nature of BSD1 protein ubiquitination. Although the LC‐MS/MS analysis on SINA1‐ubiquitinated BSD1 protein directly implicates three lysine residues (K93, K293 and K362) as the ubiquitin attachment sites by mapping of hydroxylamine‐derived peptides from ubiquitinated BSD1 protein (Figure 2G), the ubiquitination of BSD1^K93R/K293R/K362R^ mutant by SINA1 suggests other lysine sites can still be ubiquitinated by SINA1 in vitro when these three lysine residues are blocked (Figure S2C, Supporting Information). Since it has been shown that K‐to‐R mutation can cause protein conformational change, which results in exposure of nearby lysine residues for ubiquitination as well ^[^ ^29^, ^30^, ^31^, ^32^, ^33^ ^]^, future research focusing on further mutagenesis of other lysine residues will help verify this possibility. In addition, the K93R/K293R/K362R mutation likely causes a significant conformational change of BSD1 protein, which not only results in exposure of additional lysine residue(s) in the *E. coli*‐generated recombinant BSD1^K93R/K293R/K362R^ protein for ubiquitination by SINA1, but also leads to protein misfolding in plant cells, thereby disrupting the protein structure.^[^ ^34^, ^35^ ^]^ However, we cannot exclusively rule out the possibility, though less likely, that other ubiquitination sites were not identified due to the sensitivity of LC‐MS/MS analysis.

Our previous publication showed that BSD1 is able to activate the reporter lacZ gene expression in yeast,^[^ ^26^ ^]^ suggesting it may act as a transcriptional activator. However, identification of the target genes directly activated by BSD1, particularly those involved in vegetative growth, and determination of putative BSD1 binding elements are critical for understanding the signaling network controlled by the SINA1‐BSD1 regulatory module. We demonstrate that the *BRG1* gene, encoding an uncharacterized hypothetic protein, is a direct target gene of BSD1 transcription factor. Thus, BRG1 is functionally associated with SINA1 and BSD1 proteins forming a novel SINA1‐BSD1‐BRG1 regulatory cascade controlling vegetative growth in tomato. Particularly, BSD1 was able to activate the *BRG1* promoter‐driven *GUS* reporter gene via binding to a novel binding motif BBS in the *BRG1* promoter. This A/T rich so‐called BBS motif (5′‐CTTATTTC/A‐3′) has not been reported as a binding element for plant transcription factors,^[^ ^36^ ^]^ suggesting it might be specific to the BSD‐type transcription factor family and BSD1 may regulate a set of genes that possess BBS motif in their promoters. In addition to the biochemical evidence, the regulation of *BRG1* by the SINA1‐BSD1 module was verified in transgenic tomatoes whose *SINA1* or *BSD1* gene was manipulated, as manifested by dramatical down‐regulation of the *BRG1* gene in both *SINA1‐OX* and *BSD1‐KO* plants (Figure 5A). Thus, it will be interesting to determine in the future research whether other BSD‐type transcription factors can also activate *BRG1* and other genes possessing BBS motif, which will help determine whether this specific motif is a generic binding site for BSD‐type transcription factors, thereby predicting their putative target genes, regardless possessing similar or a wide range of functions.

**Figure 8:**
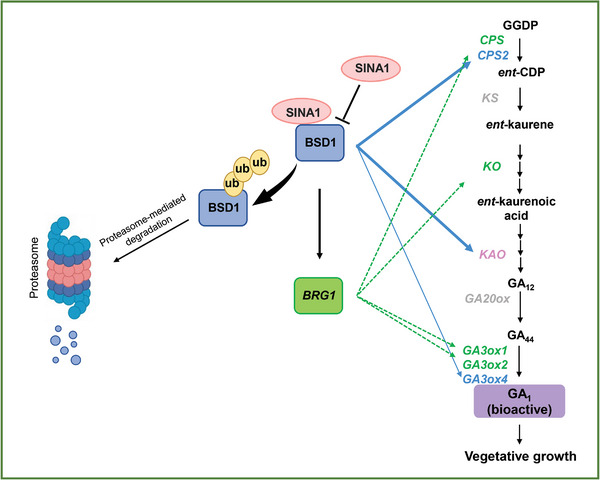
A working model for the regulation of GA‐mediated vegetative growth by a ubiquitin ligase (SINA1)‐transcription factor (BSD1) module in tomato.

The function of BRG1 protein in plant growth and development has not been studied previously. In this study we demonstrate that BRG1 plays a positive role in vegetative growth, as knockout of the *BRG1* gene resulted in dwarfism with reduced internode length (Figure 6B–E). However, the molecular role of BRG1 in the cell expansion‐associated internode elongation (Figure 6F,G) remains to be determined. Regardless the mechanistic basis by which BRG1 functions in vegetative growth, the fact that reduced GA contents in transgenic *SINA1‐OX*, *BSD1‐KO*, and *BRG1‐KO* plants with dwarfism phenotype and exogenous application of bioactive GA_3_ partially restored the vegetative growth of these dwarf plants suggests this SINA1‐BSD1‐BRG1 regulatory cascade functions through the plant growth promoting hormone GA to control vegetative growth. Although analysis of the amino acid sequence of BRG1 protein did not suggest it is a transcription factor (Figure S5A, Supporting Information), several key GA biosynthesis genes were found to be down‐regulated in *BRG1‐KO* plants (Figure 7L), implying that BRG1 is positively and indirectly involved in the regulation of the expression of GA biosynthesis genes. BRG1 may function as a novel regulator or co‐regulator for GA biosynthesis genes. For example, BRG1 may interact with other transcription factors through protein‐protein interaction to positively regulate GA biosynthesis genes. Determination of the possible interactions of BRG1 with those transcription factors or identification of additional BRG1 interacting proteins, particularly those involved in GA biosynthesis, will help address this possibility. Significantly, the BSD1‐specific BBS binding motif was found in the promoters of three GA biosynthesis genes *CPS2*, *KAO*, and *GA3ox4*. BSD1 was able to bind to the BBS‐containing promoter fragment of these genes in vitro (Figure 7H,I) and to that of *CPS2* and *GA3ox4* in vivo (Figure 7K). Additionally, BSD1 was able to activate the promoters of *CPS2*, *KAO* and *GA3ox4* genes when co‐expressed with the relevant GUS‐reporter genes in plant cells (Figure 7J). These results strongly suggest, independent of its activation on *BRG1* expression, BSD1 also directly regulates GA biosynthesis gene expression to promote GA production, thereby stimulating vegetative growth. Thus, based on our molecular, biochemical, and genetic data, we propose a model for SINA1‐BSD1‐mediated regulation of vegetative growth in tomato (**Figure** **8**). The ubiquitin ligase SINA1 negatively regulates protein level of the BSD1 transcription factor via ubiquitin‐proteasome‐mediated degradation. As a positive regulator for vegetative growth, BSD1 directly regulates three GA biosynthesis genes (*CPS2, KAO*, and *GA3ox4*) promoting GA production. Simultaneously, BSD1 also regulates the expression of *BRG1* gene that functions as a potential growth regulator involved in the expression of four GA biosynthesis genes (*CPS, KO, GA3ox1*, and *GA3ox2*). Functioning cooperatively, this SINA1 (ubiquitin ligase)‐BSD1 (transcription factor) regulatory module regulates vegetative growth involving gibberellin biosynthesis in tomato.

## 4 Experimental Section

### Plant Materials and Growth Conditions

Tomato (*Solanum lycopersicum* cv. Ailsa Craig) (AC) (from the C. M. Rick Tomato Genetics Resource Center, University of California, Davis, USA) and *Nicotiana benthamiana* were cultured in the greenhouse aided by high‐pressure sodium bulbs. The temperatures ranged from 24 to 28 °C in the daytime and 16 to 20 °C in the nighttime, respectively.

### Generation of Transgenic Tomato Plants

To generate *SINA1*‐*overexpression* (*SINA1‐OX*) plants, the full length of *SINA1* cDNA was amplified by PCR and cloned into the pBIN:c‐HA vector under the control of the CaMV 35S promoter. For the *SINA1* knockout (*SINA1‐KO*) plants, two sgRNAs targeting *SINA1* were designed according to the method described previously.^[^ ^37^ ^]^ The *BSD1*‐*OX* and *BSD1* knockdown (*BSD1*‐*Ri*) plants were described in the previous report.^[^ ^26^ ^]^ To generate the *BSD1* knockout (*BSD1‐KO*) plants, sgRNAs targeting *BSD1* were inserted into CRISPR/Cas9 vector p201N. To generate *SINA1‐OX*/*BSD1‐OX* and *SINA1‐OX*/*BSD1‐Ri* “double” transgenic tomato plants, the pCAMBIA1301 vector containing the *SINA1‐HA* construct was transformed into *BSD1‐OX* and *BSD1‐Ri* plants, respectively. To generate the *BRG1‐OX* and *‐KO* tomato plants, the full length of *BRG1* cDNA was amplified by PCR and cloned into pBIN:c‐Myc vector under the control of the CaMV 35S promoter, and two sgRNAs targeting *BRG1* were inserted into CRISPR/Cas9 vector p201N, respectively. All constructs were transformed into tomato AC cultivar by *Agrobacterium tumefaciens*‐mediated transformation. All transgenic plants were advanced to T_2_ homozygous populations and the CRISPR‐Cas9 expression cassette segregated out from the knockout mutant lines (*BSD1‐KO*, *SINA1‐KO*, and *BRG1‐KO*).

### Yeast Two‐Hybrid (Y2H) Assay

The LexA‐based Y2H system was used to determine the interactions between SINAs and BSD1. The full‐length cDNAs of *BSD1* and *SINAs* genes were cloned into the bait vector pEG202 and the prey vector pJG4‐5, respectively. The yeast (*Saccharomyces cerevisiae*) strain EGY48 harboring the *LacZα* mark gene was transformed with the bait and prey constructs in the appropriate combinations. The transformed yeast cells were streaked onto X‐Gal plates to assess the interaction between BSD1 and SINAs.

### Transient Expression Assay

For the *Agrobacterium*‐mediated transient expression in *N. benthamiana* leaves, *A. tumefaciens* cells carrying appropriate constructs were syringe‐infiltrated into *N. benthamiana* leaves. Agroinfected leaf tissues were collected 36 hours after agroinfiltration. Proteins extracted from leaf tissues were separated by SDS‐PAGE and analyzed by Western blotting using appropriate antibodies.

Polyethylene glycol (PEG)‐mediated *N. benthamiana* protoplast transient expression was conducted following previous publication.^[^ ^38^ ^]^ Typically, 200 µl of protoplast suspension (10^5^ mL^−1^) was transfected with 20 µg total DNA of appropriate plasmids. The transfected protoplasts were incubated at 22 °C for 12 hours before collection unless otherwise specified. For protein degradation assay, protoplasts were treated with 50 µm MG132 (Carbobenzoxy‐L‐leucyl‐L‐leucyl‐L‐leucinal, Sigma‐Aldrich, USA) or DMSO buffer 8 h post‐transfection with continuing incubation at 22 °C for 8 h, followed by Western blotting using appropriate antibodies.

### In Vitro Binding and Ubiquitination Assays

Recombinant proteins were expressed in *Escherichia coli* strain BL21 and purified with the ÄKTA Start Protein Purification System (GE Healthcare, Sweden) according to the manufacturer's instruction. The purified proteins were then desalted and concentrated using the Amicon Centrifugal Filters (Millipore, Ireland) with appropriate molecular weight cut‐off. The protein concentration was determined using Bradford Protein Assay (Bio‐rad, USA). For in vitro binding assay, 20 µg recombinant MBP‐BSD1‐HA protein was incubated with 15 µl anti‐HA Affinity Matrix (Roche, Germany) in 1 ml binding buffer (20 mM HEPES‐KOH, pH 7.9, 15% glycerol, 0.2 mm EDTA, 0.2% NP‐40, 1 mm DTT, and 0.1 m NaCl) for 30 min at 4 °C. The BSD1‐coupled beads were washed 3 times with wash buffer (20 mm HEPES‐KOH, pH 7.9, 15% glycerol, 0.2 mm EDTA, 0.5% NP‐40, 1 mm DTT, and 0.1 m NaCl) and the remaining active sites on the beads are blocked by 100 mg BSA to prevent nonspecific binding. To further reduce nonspecific binding, 20 µg MBP‐SINA1‐6 (MBP‐RHA1B as control) was incubated with 5 µl anti‐HA Affinity Matrix in 1 mL binding buffer for 30 min at 4 °C. After incubation, the precleared supernatant was added to the BSD1‐coupled beads and the mixture was incubated for 1 h at 4 °C. The beads were washed four times with wash buffer and heated at 95 °C in SDS loading buffer to elute bound proteins. The eluted proteins were fractionated by SDS‐PAGE and analyzed by Western blotting using appropriate antibodies.

The in vitro ubiquitination assay was conducted as described previously with minor modifications.^[^ ^39^ ^]^ 500 ng of substrate protein BSD1 was added to 30 µl ubiquitination buffer (50 mm Tris HCl, pH7.5, 2 mm ATP, 5 mm MgCl_2_, 30 mm creatine phosphate (Sigma‐Aldrich, USA), 50 ng µl^−1^ creatine phosphokinase (Sigma‐Aldrich, USA) containing 40 ng His‐E1 (AtUBA1), 100 ng His‐E2 (SlUBC12), 1 µg E3 (MBP‐SINA1‐6, MBP‐SINA1^C63S^ or MBP alone) and 2 µg ubiquitin‐Flag (Sigma‐Aldrich, USA)). The reaction mixtures were incubated at 30 °C for 2 h. Reactions were then terminated by the addition of 7.5 µl 5×SDS loading buffer and heated to 95 °C for 5 min, followed by protein separation by SDS‐PAGE and analyzed by Western blotting using appropriate antibodies.

### Mapping of Ubiquitination Sites by Liquid Chromatography‐Tandem Mass Spectrometry

To map the ubiquitination sites of BSD1, 5 µg substrate (MBP‐BSD1‐HA) was subjected to in vitro ubiquitination assay and the ubiquitinated BSD1 was separated by SDS‐PAGE gel. The bands (0.5 cm) above the BSD1 band were excised, and in‐gel digested by trypsin at 37 °C for 16 h.

An Orbitrap Fusion Tribrid mass spectrometer coupled with an UltiMate 3000 RSLCnano UHPLC System (Thermo Scientific) was used for LC‐MS/MS analysis of the tryptic peptides. Full MS (250–1800 m z^−1^) was detected in Orbitrap with the resolution of 60 K. Then MS1 ions were isolated by Quadrupole with the isolation window 1.6 for collision‐induced fragmentation, and the resulting MS2 ions were detected by Linear Ion Trap. The LC was at a flow rate of 0.3 µL min^−1^ for 45 min with the following gradient (solvent A, 0.1% formic acid in water; solvent B, 0.1% formic acid in 80% acetonitrile): 2–4% B in 4 min, 4–32% B over 30 min, and then ramped up to 95% B in 2 min, held at 95% B for 2 min and returned to 2% B in 1 min. The raw files were searched using Mascot (version 2.2, Matrix Science) with the Tomato protein fasta database downloaded from UniProt (on April 5, 2024). The parameters were set as: precursor mass tolerance 10 ppm, fragment mass tolerance 0.8 Da, trypsin with one missed cleavage, oxidation (M), and GlyGly (K) as dynamic modifications. The three ubiquitinated peptides of BSD1 were identified only in the samples with both E3 ligase and substrate present, but not in other controls. The identified ubiquitinated peptides and each ubiquitination site assignment were confirmed by manual inspection of the precursor mass, internal fragments, as well as b and y ions.

### Cell‐Free Protein Degradation and Inhibition Assay

The protein inhibition assay was carried out as reported previously.^[^ ^40^ ^]^ Briefly, 1 g fresh leaf and lateral bud tissues from one‐month‐old *SINA1‐OX* transgenic plants were ground into powder using liquid nitrogen, followed by the extraction of total proteins with extraction buffer (25 mm Tris/HCl, pH 7.5, 10 mm MgCl_2_, 10 mm NaCl, 4 mm PMSF, 5 mm DTT, and 10 mm ATP). 1 µg recombinant MBP‐BSD1‐HA protein was incubated with equally divided supernatant with or without MG115. Protein levels were determined by Western blotting with anti‐HA antibody, Image J software (http://imagej.nih.gov/ij/) was used to quantify the signal intensity.

### Validation of Ubiquitination Sites

Single (K93R, K293R, K362R) and triple (K93R/K293R/K362R) mutations were introduced into the BSD1 coding sequence using overlapping PCR. These mutants were cloned into the pBIN‐C2‐HA vector for transient expression in *N. benthamiana* leaves. Gene expression and protein levels were assessed by RT‐PCR and Western blotting, respectively. The BSD1 triple mutant with HA tag was inserted into the pMAl‐C2 vector and expressed in *E. coli* to generate protein purification, which was used for in vitro ubiquitination assay.

### GUS Staining Assay

The promoter regions of *BRG1* (*Solyc07g054310*) and the other three candidates were amplified and inserted into the upstream of the *GUS* gene in pBI35Smini vector as described previously.^[^ ^41^ ^]^ After Agrobacterium‐mediated transient expression in *N. benthamiana* leaves for 2 days, staining assay was conducted by incubation of infected leaves with X‐gluc buffer at 37 °C overnight, followed by de‐staining with 95% ethanol.

### ChIP Assay

Leaves of *BSD1‐OX‐HA* and AC tomato plants were harvested for the ChIP experiments using anti‐HA affinity matrix (Roche, USA) or negative control anti‐Myc affinity matrix (Chromotek, Germany) following the procedures described previously.^[^ ^42^ ^]^ Briefly, 1 g leaf tissue ground in liquid nitrogen was resuspended in 5 ml cold lysis buffer (20 mm Tris HCl, pH7.5, 25% Glycerol, 20 nm KCl, 2 mm DTA, pH8.0, 2.5 mm MgCl_2_, 250 mm Sucrose) and crosslinked with 1% formaldehyde rotated at 4 °C for 20 min. Crosslinking was terminated by 0.125 m glycine. Chromatin was extracted and sonicated for the preparation of DNA associated with chromatin. BSD1‐associated DNA enriched with anti‐HA and anti‐Myc (served as a control) affinity matrixes was recovered for qPCR using BSD1 promoter‐specific primers. qPCR primers are listed in Table S2 (Supporting Information) and three biological replicates were performed for ChIP‐qPCR assay using an ABI 7300 Real‐time PCR system (Applied Biosystems, USA).

### Electrophoresis Mobility Shift Assay (EMSA)

The recombinant His‐BSD1 protein was purified from *E.coli* as described above. EMSA was conducted using the LightShift chemiluminescent EMSA kit (Thermo Fisher Scientific, USA). Briefly, the sense and anti‐sense oligonucleotides were biotinylated with the Biotin 3′ End DNA labeling (Thermo Fisher Scientific, USA) and annealed to double‐strand oligonucleotides. The labeled oligonucleotides were incubated with His‐BSD1 protein based on the manufacturer's instruction. Unlabeled oligonucleotides were included for the competition assay. After 20 min of incubation at room temperature, the samples were separated with a 10% native DNA polyacrylamide gel, followed by electrophoretic transfer to Hybond‐N nylon membrane (Amersham Pharmacia Biotech, USA). The membrane was crosslinked by UV light for 15 min, followed by chemiluminescence detection.

### Histological Analysis

Plant materials, fixed in the fixing solution (formalin: acetic acid: 70% ethanol (1:1:18)), were embedded in paraffin (Thermo Fisher Scientific, USA). 20 µm sections were cut with SENIOR ROTARY MICROTOME (Radical Scientific Equipments, India) and stained with toluidine blue dye (Thermo Fisher Scientific, USA). The stained sections were subjected to microscopy with a fluorescent microscope (Leica Microsystems, Wetzlar, Germany).

### Subcellular Localization of the BRG1 Protein

Protoplasts isolated from 5‐week‐old *N. benthamiana* leaves were transformed with *pTEX:GFP* and *pTEX:GFP‐BRG1* plasmids, respectively. After overnight incubation, the transformed cells were subjected to microscopy with fluorescent microscope (Leica Microsystems, Germany) to capture the GFP signal.

### Quantification of Gibberellin in Plants

The third internode stem tissues, from top to bottom, were harvested from tomato plants and the endogenous gibberellin contents were determined as described.^[^ ^43^ ^]^ Briefly, 5 µl of 1 ng ml^−1^ deuterium‐labeled GA_1_ (acted as internal standard; Olchemim Ltd., Czechoslovakia) was added to 50–100 mg ground tissue with liquid nitrogen. The mixture was dissolved in 600 µl of extraction solvent (75% MeOH, 20% H_2_O, and 5% FA) and shaken at 500 rpm for 2 h, followed by overnight extraction without shaking in cold room. The reaction mixture with 600 (300×2) µl of MeOH was centrifugated (10000 × g) at 4 °C for 10 min and the supernatant was dried with nitrogen gas. The dried sample was then re‐dissolved in ddH_2_O (pH 2.5), purified three times with ethyl acetate and dried one more time for derivatization. For the *N*‐(3‐dimethylaminopropyl)‐*N'*‐ethylcarbodiimide hydrochloride (EDC) labeling, the dried sample (ultrasonicated for 5 min prior to derivatization) and a series of GA standards dried with nitrogen gas were incubated with 50 µl of 40 mM EDC at 35 °C with shaking at 750 rpm for 1.5 h. After incubation, the reaction mixture was centrifuged at 12 000 rpm for 5 min and the supernatant was ready for GA_1_ determination by LC‐MS/MS.

LC‐MS/MS analysis was conducted using a Waters Acquity UPLC interfaced with a Waters TQ MS mass spectrometer. A Waters Acquity UPLC BEH C18 column (2.1×50 mm 1.7 um, P/N 186002350) was used for the separation of derivatized gibberellic acids. Mobile phase solvent A was 0.1% formic acid in water and solvent B was 0.1% formic acid in acetonitrile. The analytical column was maintained at 40 °C and the injection volume was 10 µl. Initial solvent composition was 99% A and 1% B at a flow rate of 0.2 ml min^−1^. Analyte separation and elution were accomplished by increasing the composition of solvent B to 4% from initial injection to 1 min, then increasing to 6% at 2 min, increasing to 9% at 5 min, increasing to 12% at 9 min, increasing to 34% at 21 min, increasing to 50% at 25 min, and finally increasing to 100% at 27 min. The composition was held at 100% B for 1 min, returned to 1% B over the next 4 min, and allowed to equilibrate at 1% B for 3 min prior to the next injection.

MS data were acquired in positive ion mode using a multiple reaction monitoring method with a capillary voltage of 3.2 kV, source temperature of 150 °C, desolvation temperature of 350 °C, and desolvation gas flow of 650 L/h. The cone voltage was 28 V for all analytes and standards and the collision energy was 34 V for EDC‐gibberellic acid A1 and d4‐A1. The MRM transition for EDC‐gibberellic acid A1 was 504.3 > 388.2, 508.3 > 392.2 for EDC‐gibberellic acid d4‐A1. Dwell time was 0.370 s for each analyte and standard. MS data was analyzed using the TargetLynx add‐on software to Waters MassLynx software ver. 4.1.

### Hormone Treatment

For the hormone treatment assay, two‐week‐old tomato seedlings were sprayed twice a week with GA_3_ (Sigma‐Aldrich) at a concentration of 20 µg ml^−1^ containing 0.025% (v v^−1^) Triton X‐100 as surfactant, and control plants were sprayed with the same solvent without GA_3_. After spraying 4 times, the plant height was determined. Statistical significance was determined using a *t*‐Test.

### Accession Numbers

Sequence data from this article can be found in SGN database (Sol Genomics Network) under accession numbers: *BSD1*, *Solyc04g077600*; *SINA1*, *Solyc01g006190*; *SINA2*, *Solyc01g096020*; *SINA3*, *Solyc05g050580*; *SINA4*, *Solyc06g051980*; *SINA5*, *Solyc03g083270*; *SINA6*, *Solyc10g009420*; *BRG1*, *Solyc07g054310*; *Actin‐41*, *Solyc04g011500*.

## Conflict of Interest

The authors declare no conflict of interest.

## Author Contributions

Y.Y., Y.F., and L.H. contributed equally to this work. Y.Y., Y.F., L.H., Y.L., and F.X. conceived and designed the experiments, Y.Y., Y.F., and L.H. generated transgenic plants, Y.Y., Y.F., L.H., and X.N. performed the phenotypic experiments. H.L. and M.G. conducted a subcellular localization assay, C.X. and C.Z. did RNAseq analysis, B.T. and S.C. conducted LC‐MS/MS assay and identified the ubiquitination sites, Y.Y., Y.F., H.L., and L.H. performed biochemical assays, Y.Y., Y.F., L.H., C.R., Y.L., and F.X. analyzed the data, Y.Y., Y.F., C.R. and F.X. wrote the paper.

## Supporting information

Supporting Information

Supporting Information

## Data Availability

The data that support the findings of this study are available from the corresponding author upon reasonable request.
