# Supplementary material for: The SINA1‐BSD1 Module Regulates Vegetative Growth Involving Gibberellin Biosynthesis in Tomato
Source: Adv Sci (Weinh). 2024 Aug 27;11(40):2400995. doi: 10.1002/advs.202400995 (PMC11633369; doi:10.1002/advs.202400995)
Supplement: Supplementary file 1 — Supporting Information [file ADVS-11-2400995-s002.docx]

Supporting Information

Title The SINA1-BSD1 Module Regulates Vegetative Growth Involving Gibberellin Biosynthesis in Tomato

Yulin Yuan, Youhong Fana, Li Huang, Han Lu, Bowen Tan, Chao Xia, Xiangli Niu, Sixue Chen, Mingjun Gao, Cankui Zhang, Yongsheng Liu, and Fangming Xiao*


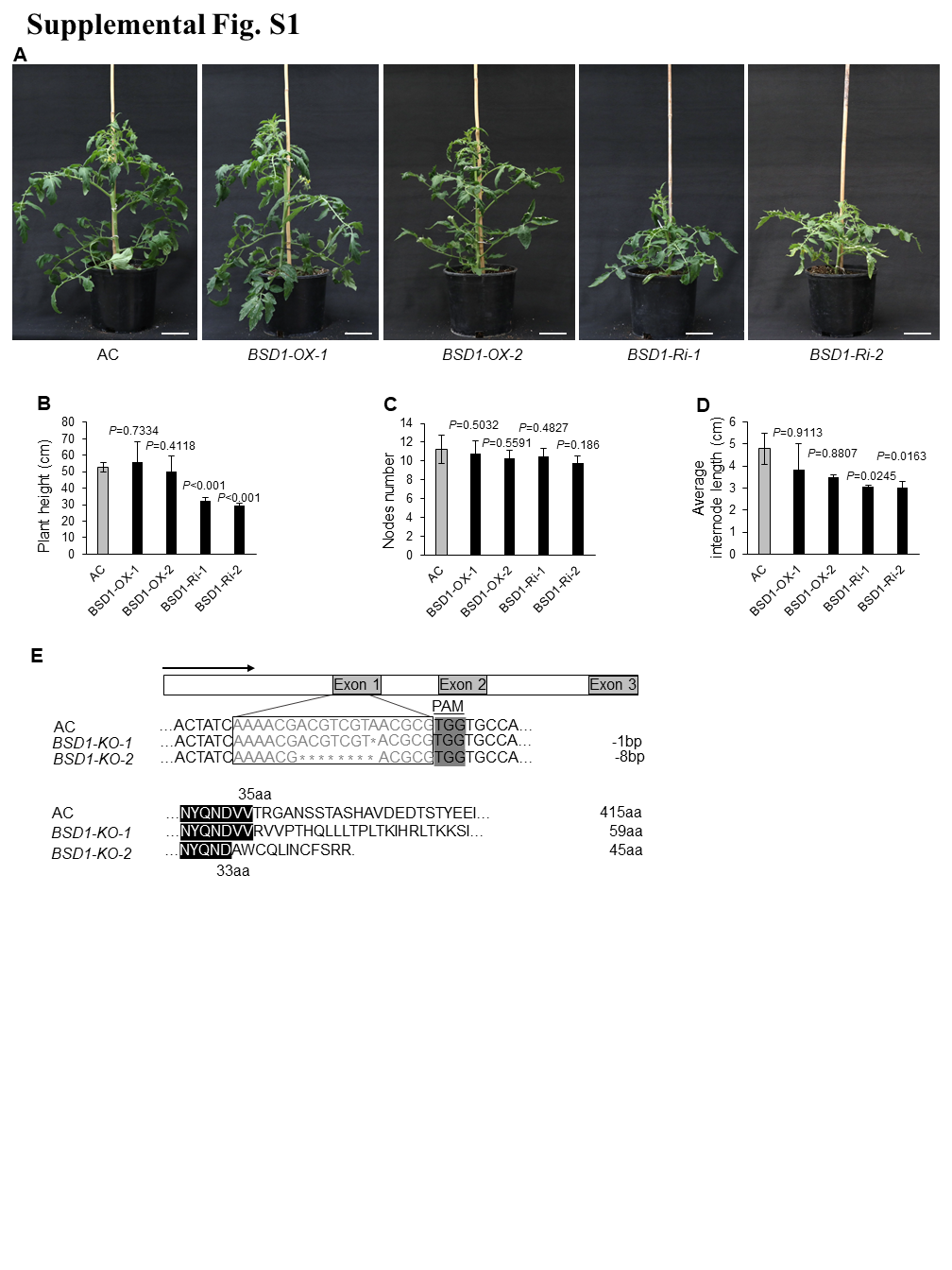


**Figure S1. BSD1 is required for vegetative growth in tomato.** A) The morphologic appearance of representative six-week-old AC, *BSD1-OX* and *BSD1-Ri* plants. Scale bar, 10 cm. B-D) Quantification of plant height (B), node number (C) and average internode length (D) of plants in (A). N=4. *P v*alues are indicated in the graph. E) CRISPR-Cas9-based generation of *BSD1* knockout transgenic tomato plants. The top panel indicates the guide RNA sequence targeting the first exon region of *BSD1*. TGG highlighted in grey represents protospacer adjacent motif (PAM) sequences. The black arrow indicates the direction of transcription. Nucleotide alignment (middle panel) and amino acid alignment (bottom panel) indicate the distinct deletion mutations in two *BSD1-KO* lines. Data are means ± SD; **p*<0.05, two-tailed T tests. All experiments have been repeated at least twice with similar results.


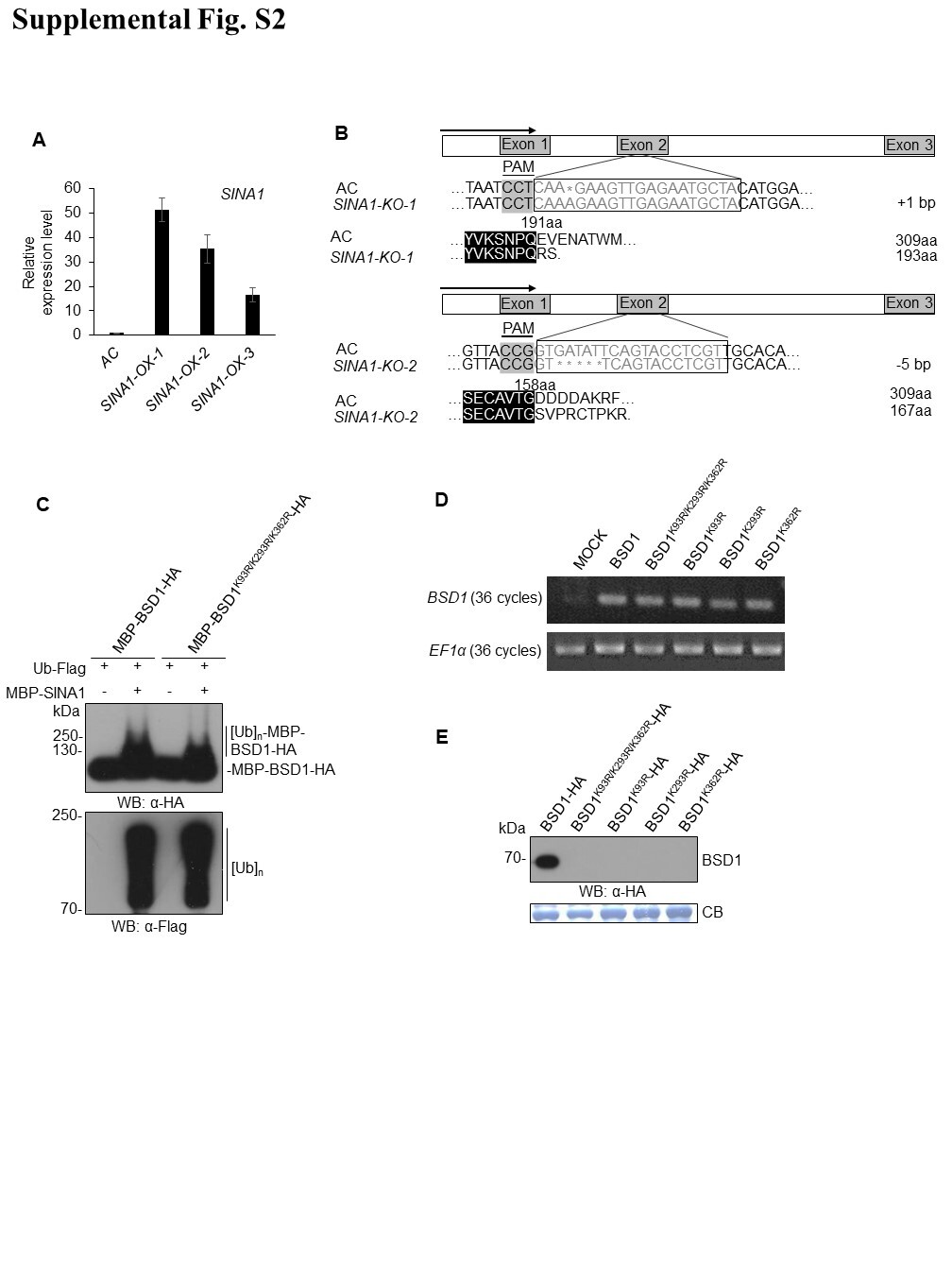


**Figure S2.** **Generation of *SINA1* overexpression and knockout transgenic tomatoes.** A) The relative expression levels of *SINA1* in AC and *SINA1-OX* plants. B) CRISPR-Cas9-based generation of *SINA1* knockout transgenic tomato plants. Data are means ± SD. C) *In vitro* ubiquitination of BSD1 and BSD1^K93R/K293R/K362R^ mutant by SINA1. The smear banding pattern recognized by anti-HA antibody indicates only marginal decrease of SINA1-mediated ubiquitination of BSD1^K93R/K293R/K362R^ mutant compared to that of the WT BSD1 protein (top panel). Anti-Flag indicates total ubiquitination catalyzed by SINA1 (bottom panel). D) Confirmation of the expression of the BSD1 and derived mutant constructs in plant cells. WT BSD1 and derived mutants driven by the CaMV 35S promoter were transiently overexpressed in *N. benthamiana* leaves and their expression levels were determined by RT-PCR. *EF1α* was used as an internal control. E) K93, K293 and K362 are essential for BSD1 protein stability. Western blotting indicates that the epitope-tagged WT BSD1-HA protein, but not the mutant proteins (BSD1^K93R^-HA, BSD1^K293R^-HA BSD1^K362R^-HA and BSD1^K93R/K293R/K362R^-HA), is detectable by anti-HA antibody when transiently expressed in *N. benthamiana* leaves. CB verifies equal loading of proteins. All experiments have been repeated at least twice with similar results.

**
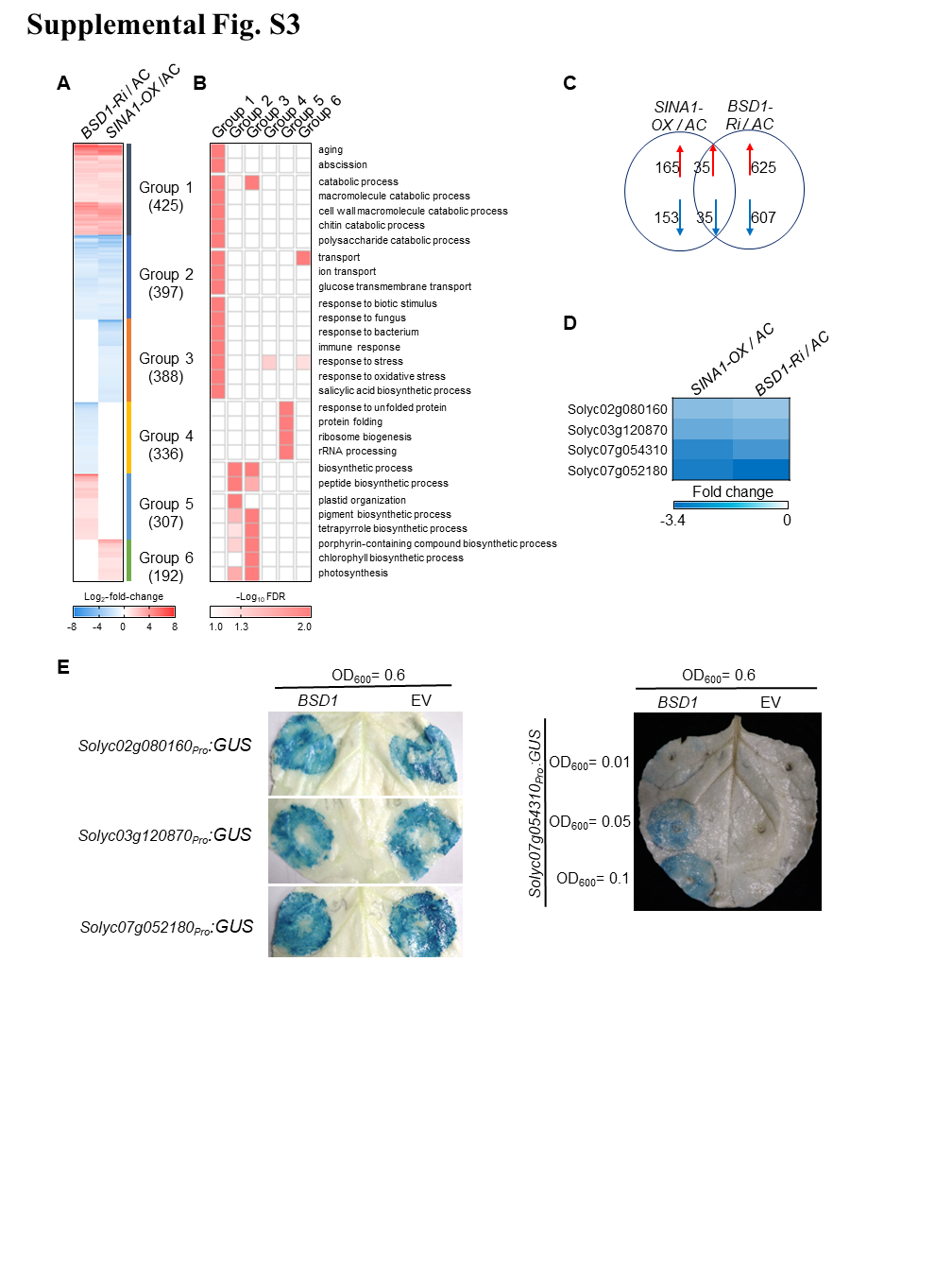
Figure S3. BSD1 activates the expression of *BRG1* gene.** A) Six major groups of differentially expressed genes (DEGs). The numbers of DEGs in each group are denoted in parentheses. Red and blue colors represent up- and down-regulation in the transgenic leaves compared with the AC leaves, with the shade indicating fold change as shown in the color bar. B) Enriched GO biological process terms in genes in Group 1-6. Red color represents GO terms that are significantly overrepresented (Benjamini-Hochberg FDR corrected *P* value<0.05), with the shade indicating significance as shown in the color bar. Shown are a limited number of representative GO terms (see Supplemental Data Set S1 for full results). C) Venn diagram of differentially expressed genes (fold change >2) shared between *SINA1-OX* and *BSD1-Ri*. The arrows in red and black indicate the up or down-regulated genes, respectively. D) Heatmap of four genes showing differential expression in *SINA1-OX* vs AC and *BSD1-Ri* vs AC. The shade indicates fold change as shown in the color bar. E) The *BRG1* (*Solyc07g054310*) promoter is activated by BSD1. The promoters of *BRG1* (right panel) and the other three potential genes (left panel) fused with *GUS* reporter gene were co-expressed with BSD1 or empty vector (EV) in *N. benthamiana* leaves at different inocula (indicated as the value of OD_600_) via *Agrobacterium*-mediated transient expression. Two days after agrobacterial infiltration, the leaves were stained using X-Gluc and de-stained with 95% ethanol prior to photograph.


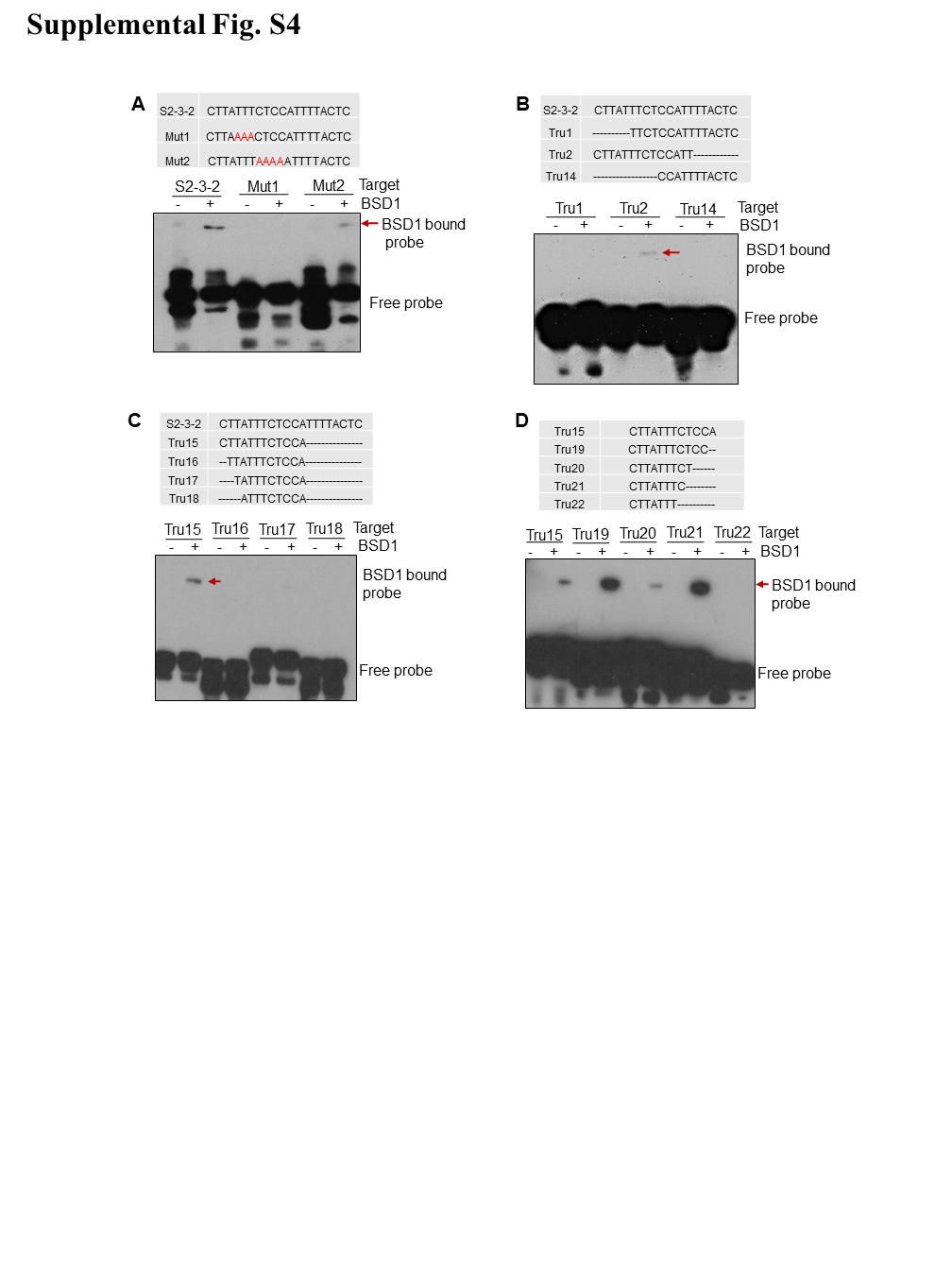


**Figure S4. BSD1 regulates *BRG1* via binding to the BBS motif.** EMSA assay using serial truncations determines that BSD1 directly binds to the BBS motif in *BRG1* promoter. A) S2-3-2 is the specific region bound by BSD1. Two S2-3-2 mutant probes (Named as Mut1 and 2) were incubated with or without BSD1 protein and BSD1 was not able to bind to Mut1. The nucleotides highlighted in red represent mutations. B) The N-terminus of S2-3-2 is essential for BSD1 binding. Truncations (Tru1, 2 and 14) missing either the N-terminus or C-terminus of S2-3-2 were used to identify the direct binding site. C, D) BSD1 binds to the BBS motif. Serial truncation mutants with single nucleotide deletion were incubated with or without BSD1 protein. The minimal region sufficient for BSD1 binding was 5’-CTTATTTC-3’ and designated as BBS (standing for BSD1 binding site) motif. All experiments have been repeated at least twice with similar results.


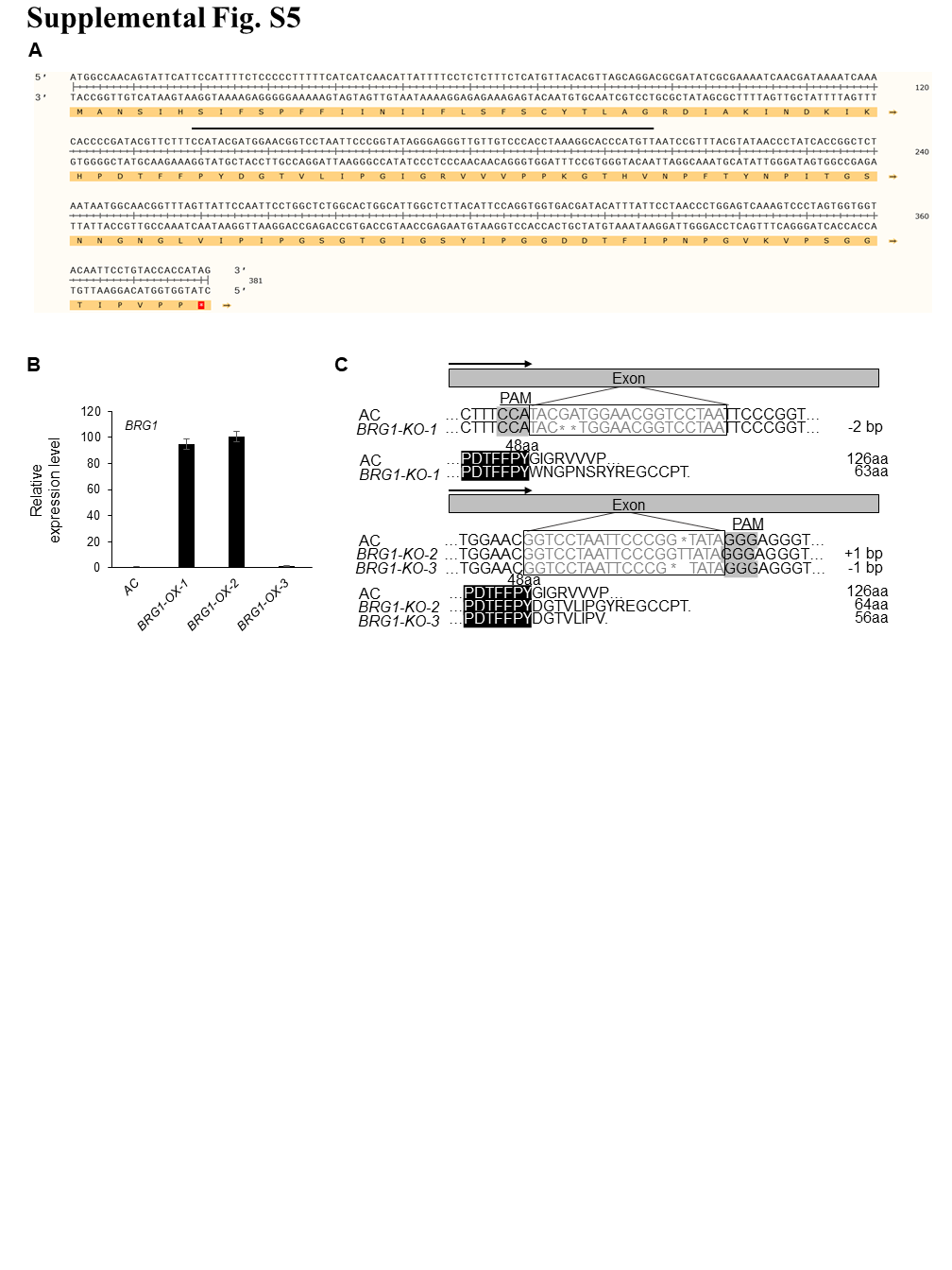


**Figure S5. Generation of *BRG1* overexpression and knockout transgenic tomatoes.** A) Sequences of the *BRG1* cDNA and encoded protein with a predicted transmembrane domain being underlined. B) The relative expression levels of *BRG1* in AC and *BRG1-OX* plants. C) CRISPR-Cas9-based generation of *BRG1* knockout transgenic tomato plants.

**
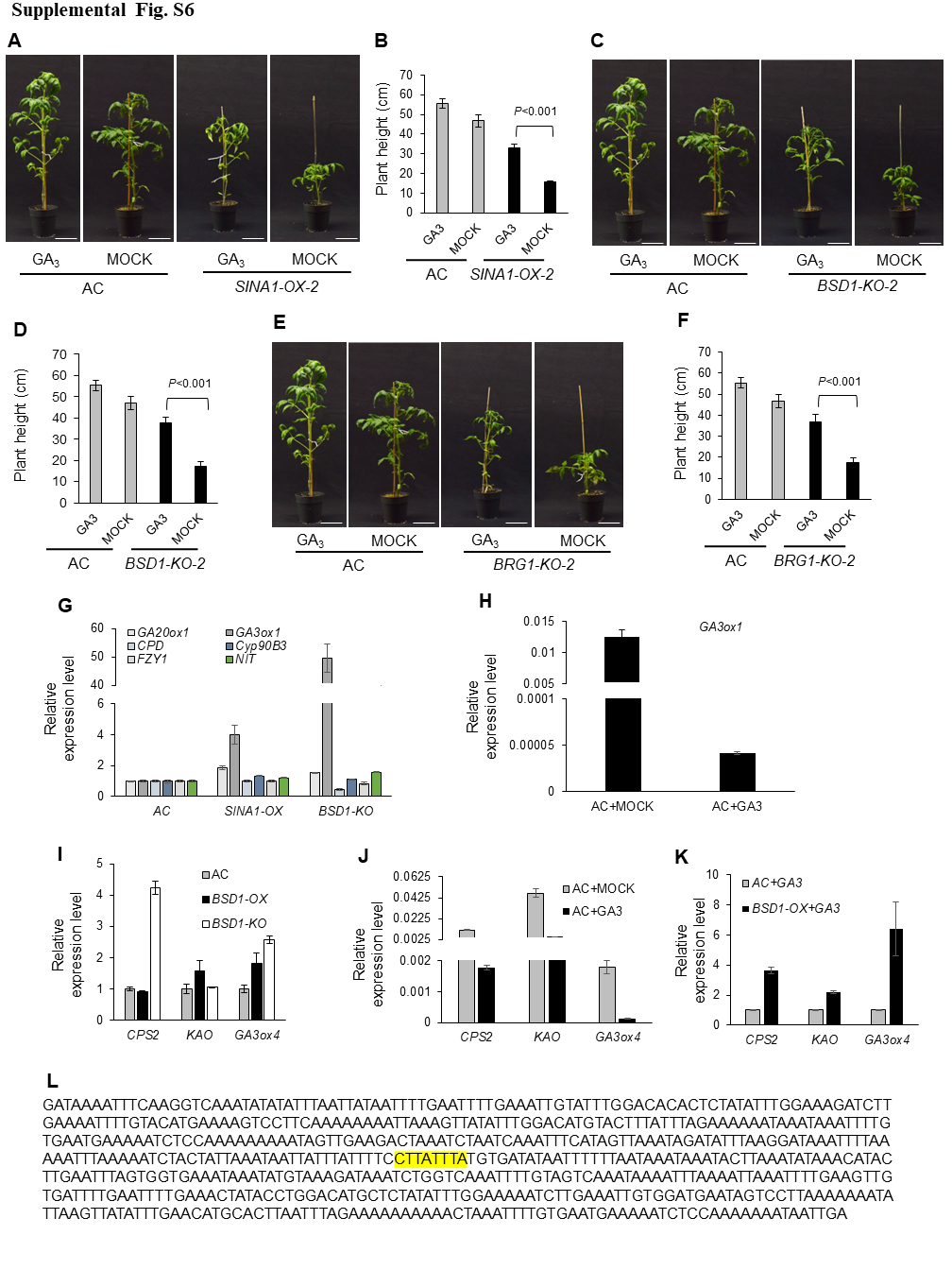
**

**Figure S6.** **The SINA1-BSD1 module regulates vegetative growth through modulating GA biosynthesis genes.** A-F) Additional lines of *SINA1-OX*, *BSD1-KO and BRG1-KO* treated with GA_3_. Note that the AC control images in (A, C, E) are identical and from the same experiment.

G, H) The SINA1-BSD1 module is involved in the regulation of GA biosynthesis. (G) The expression of genes related to GA, BR and auxin hormones, including two GA-related genes (*GA20ox1, GA3ox1*), two BR-related genes (*CPD*, *CYP90B3*) and two auxin-related genes (*FZY1*, *NIT*) in the 3^rd^ stem tissues of six-week-old *SINA1-OX* and *BSD1-KO* plants were measured. (H) *GA3ox1* gene undergoes feedback regulation. AC tomato internodes with or without GA_3_ treatment were harvested to determine the expression of *GA3ox1* gene. I-K) BSD1 positively induces the expression of *CPS2, KAO* and *GA3ox4* genes. AC, *BSD1-OX* and *BSD1-KO* exhibit variations in *CPS2, KAO* and *GA3ox4* gene expression (I). *CPS2, KAO* and *GA3ox4* genes are subjected to GA-mediated feedback regulation (J). To accurately determine the effect of *BSD1* overexpression on the expression of these genes, we examined the expression of three genes in both AC and *BSD1-OX* lines after GA_3_ treatment. *CPS2, KAO* and *GA3ox4* genes are upregulated by BSD1 (K). L) Sequences of the *KAO* promoter region flanking the BBS motif. Long stretches of identical nucleotides (particularly Adenines [As] and Thymines [Ts]) are present within ±300 bp of the BBS motif. The BBS motif is highlighted in yellow. All experiments have been repeated at least twice with similar results.

**Supplemental Table S1.** RNA-seq analysis of *SINA1-OX, BSD1-Ri* and AC leaves.

**Supplemental Table S2.** List of primers used in this study.

**Supplemental Data Set S1.** GOBPs enriched by genes in Group1-6. Groups of DEGs categorized by their differential expression patterns in the comparisons of *BSD1-Ri* and *SINA1-OX* versus AC.
